# Experimental verification of generalized eigenstate thermalization hypothesis in an integrable system

**DOI:** 10.1038/s41377-022-00887-5

**Published:** 2022-06-28

**Authors:** Qin-Qin Wang, Si-Jing Tao, Wei-Wei Pan, Zhe Chen, Geng Chen, Kai Sun, Jin-Shi Xu, Xiao-Ye Xu, Yong-Jian Han, Chuan-Feng Li, Guang-Can Guo

**Affiliations:** 1 CAS Key Laboratory of Quantum Information, University of Science and Technology of China, Hefei, 230026 China; 2 CAS Center for Excellence in Quantum Information and Quantum Physics, University of Science and Technology of China, Hefei, 230026 China; 3 Hefei National Laboratory, University of Science and Technology of China, Hefei, 230088 China

**Keywords:** Quantum optics, Single photons and quantum effects

## Abstract

Identifying the general mechanics behind the equilibration of a complex isolated quantum system towards a state described by only a few parameters has been the focus of attention in non-equilibrium thermodynamics. And several experimentally unproven conjectures are proposed for the statistical description of quantum (non-)integrable models. The plausible eigenstate thermalization hypothesis (ETH), which suggests that each energy eigenstate itself is thermal, plays a crucial role in understanding the quantum thermalization in non-integrable systems; it is commonly believed that it does not exist in integrable systems. Nevertheless, integrable systems can still relax to the generalized Gibbs ensemble. From a microscopic perspective, understanding the origin of this generalized thermalization that occurs in an isolated integrable system is a fundamental open question lacking experimental investigations. Herein, we experimentally investigated the spin subsystem relaxation in an isolated spin–orbit coupling quantum system. By applying the quantum state engineering technique, we initialized the system with various distribution widths in the mutual eigenbasis of the conserved quantities. Then, we compared the steady state of the spin subsystem reached in a long-time coherent dynamics to the prediction of a generalized version of ETH and the underlying mechanism of the generalized thermalization is experimentally verified for the first time. Our results facilitate understanding the origin of quantum statistical mechanics.

## Introduction

**Fig. 1:**
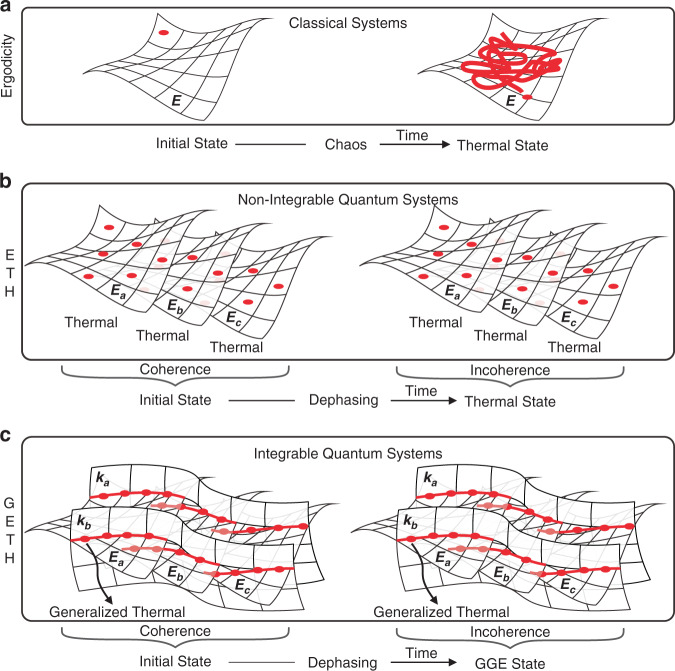
Mechanism of the thermalization and the generalized thermalization. An illustration of the following three points: **a** the ergodicity in classical systems, **b** the ETH supporting the thermalization in non-integrable quantum systems^6^, and **c** the GETH supporting the generalized thermalization in integrable quantum systems

Statistical mechanics predicts that an isolated classical system relaxes to a thermal state which is determined only by its energy and independent of the other details of its initial conditions^1–3^. This “universality” of thermalization has been well-understood by utilizing ergodicity in classical theories (Fig. 1a). Unfortunately, due to the general absence of ergodicity in quantum systems, this successful theoretical framework cannot be directly applied to the quantum scenarios^4,5^. Therefore, the origin of thermalization in an isolated quantum system must fundamentally differ from that of its classical counterpart.

For an isolated quantum system, its thermalization is usually revealed by the long-time average of the observables of interest, which can also be directly calculated from an ensemble of its energy eigenstates^6^. Further, since the expectation of the observables in a thermal state can be obtained by averaging on a uniformly distributed micro-canonical ensemble (ME), which is constructed within the constant-energy manifold and independent of the initial details, this alleged “diagonal ensemble”, which depends on the details of the initial energy distributions, is explicitly conflicted. To explain the elimination of the initial information in the thermalization process of an isolated quantum system, some new insights into the quantum theory are needed. Actually, understanding the origin of the “universality” of the equilibrium states in isolated quantum systems has become one of the central problems in the subject of quantum non-equilibrium physics^7^.

Among the numerous efforts, the eigenstate thermalization hypothesis (ETH)^6,8,9^, which states that thermalization occurs at the level of a single energy eigenstate of a given Hamiltonian (Fig. 1b), plays a key role in explaining the quantum thermalization. More concretely, based on ETH, if the expectation of a local observable in the energy eigenstates continuously changes along with the energy, its long-time average for any superposition state of the eigenstates with similar energy is independent of the details of the initial states, and this can be efficiently calculated by averaging in the corresponding ME. It is generally believed that generic quantum many-body systems, except some special ones with many-body localization^10,11^, should satisfy the ETH and can be thermalized^6,12–21^.

However, extensively theoretical investigations, ranging from one-dimensional hard-core bosons to transverse-field Ising chain^22–27^, have confirmed that the celebrated ETH is broken down in such integrable quantum systems, which have a non-trivial set of conserved quantities and cannot consequently thermalize. Nevertheless, the integrable systems can still relax to the maximum entropy state with given constants of motion, which is generally called the generalized Gibbs ensemble (GGE)^22^, and exhibit the generalized thermalization^27,28^. Admittedly, the GGE is not a thermal state and consequently cannot be directly understood using the ETH. However, its underlying mechanism has been found to be explicable by employing the generalized version of ETH (GETH)^28^, in which the conventional energy eigenstates are upgraded to the mutual ones of the Hamiltonian and a set of conserved quantities of the quantum system (Fig. 1c). Although significant developments have been achieved by applying quantum simulation^29–37^, even the GGE has been observed in a degenerate one-dimensional Bose gas^38^. The GETH, previously introduced to explain the generalized thermalization of integrable models, still lacks a direct experimental verification because of the challenges in generating the given superposition states of the system’s mutual eigenstates^39^ and requiring long-time coherent dynamics.

In this study, in an integrable quantum system with spin–orbit coupling, we experimentally verified the GETH from the spin relaxation by applying an asymptotic method. The experiment was carried out in our large-scale photonic discrete-time quantum walk (QW)^40^. The system can be well-isolated and consequently can maintain a long-time coherent evolution^41,42^ to explore the pure state quantum statistical mechanics^43^. More importantly, as the key techniques for investigating the GETH, both the ability to engineer the initial states^44,45^ and the full reconstruction of the spinor eigenvectors of a given Hamiltonian^40^ are accessible in our framework. Benefiting from these unique techniques, we monitored the spin subsystem relaxation after preparing the whole system with different distribution widths on the mutual eigenstate basis of the conserved quantities. Our experimental results demonstrated that the long-time-averaged spin state from the superposition of the mutual eigenstates with the similar conserved quantities can be predicted by the generalized ME, and explicitly support the GETH in the integrable systems.

## Theoretical background

### The model of QW

**Fig. 2:**
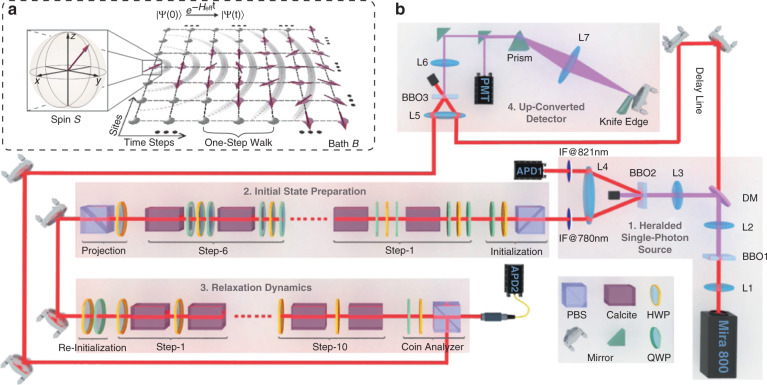
Quantum walk model and the experimental setup. **a** A diagram of an isolated QW for investigating the generalized thermalization, which contains two parts: the discrete infinite lattice ${{{\mathcal{B}}}}$ and spin (coin) subsystem ${{{\mathcal{S}}}}$, coupled by the spin–orbit interaction. **b** The experimental realization of QW involves the following four modules. (1) Pairs of photons are generated via the spontaneous parametric down-conversion (SPDC) in the BBO2 crystal, where a photon from each pair is directly detected by the APD1 as a trigger and the other signal one as the walker is sent to the subsequent module. (2) First, a localized state is initialized via a PBS-HWP-QWP setting. Second, the signal photons go through at most six steps of walk with each step involving two SU(2) coin tossings achieved by the combination QWP-HWP-QWP and two shift operators achieved by the calcite crystals. An arbitrary Gaussian wavepacket over the lattice space can be obtained after projecting the coin on an unbiased basis $\left| + \right\rangle$ using an HWP and PBS. (3) After re-initialization of the coin state (here the time step is also re-initialized as *t* = 0) and sending the signal photons into a 10-step walk whose coin-tossing rotations along the y-axis and achieved by utilizing the HWP, the relaxation evolution of the coin state can be monitored through a coin analyzer. (4) The time-evolving probability distributions, i.e., the pulse train of signal photons with a time interval of approximately 5 ps, can be analyzed by utilizing the up-converted detector, which comprised a frequency upconversion in the BBO3 crystal, a 4-f system for filtering the spectrum, and a PMT detector. The 4-f system includes a prism for introducing dispersion, a knife-edge for blocking the noise photons whose wavelength is longer than 395 nm, and a mirror reflecting the up-converted signal photons to the PMT. List of the abbreviations: lens (L); β-BaB_2_O_4_ (BBO); avalanche photodiode detector (APD); dichroic mirror (DM); polarization-dependent beam splitter (PBS); half-wave plate (HWP); quarter-wave plate (QWP); photomultiplier tube (PMT). Detailed descriptions are provided in the Methods

Herein, we consider a discrete-time version of QW^46–48^ that describes the coherent hopping of a single microscopic particle on a discrete infinite lattice, where a quantum coin determines the transition amplitudes. Consequently, the system contains two interacted subsystems: the coin (spin) and lattice, as shown in Fig. 2a. We take the lattice as bath ${{{\mathcal{B}}}}$ and the spin as the subsystem of interest ${{{\mathcal{S}}}}$ (i.e., the local observables of interest are on the spin), whose relaxation is investigated to reveal the equilibration and generalized thermalization.

The dynamics of the whole system can be described as a stroboscopic operation *U*^*t*^ with *U* representing a single step of walk and the time $t \in {\Bbb Z}$ being in a discrete scenario. Effectively, the unitary time evolution can be expressed as $e^{i{{{\mathcal{H}}}}_{{{{\mathrm{eff}}}}}t}$ in terms of an effective Hamiltonian ${{{\mathcal{H}}}}_{{{{\mathrm{eff}}}}}$. For a split-step version^49^, the effective Hamiltonian reads (see Methods): ${{{\mathcal{H}}}}_{{{{\mathrm{eff}}}}}\left( {\theta _1,\theta _2} \right) = {\int}_{ - \pi }^\pi {dk\left[ {E\left( k \right){{{\mathbf{n}}}}_{{{\mathcal{H}}}}\left( k \right) \cdot \vec \sigma } \right]} \otimes \left| k \right\rangle \left\langle k \right|$, where *θ*_1_ and *θ*_2_ are the control parameters of the coin-tossing operators, *E*(*k*) gives the (quasi-)energy band with ${{{\mathbf{n}}}}_{{{\mathcal{H}}}}\left( k \right)$ denoting the corresponding spinor eigenvector for the (quasi-)momentum *k*, and $\vec \sigma = \left( {\sigma _x,\sigma _y,\sigma _z} \right)$ is the Pauli matrix. The interchange of energy between subsystem ${{{\mathcal{S}}}}$ and bath ${{{\mathcal{B}}}}$ is implemented via the spin–orbit coupling. This typical integrable model has been extensively applied to investigate the general properties of the spin-orbital coupling system, especially its topological characters^50^. Herein, we explore the GETH and generalized thermalization in this model.

### Equilibration of the spin subsystem

As shown in Supplementary Section A, the long-time-averaged state of the spin subsystem in the QW can always relax to a steady state^51,52^: $\rho _{{{{\mathrm{st}}}}} = \mathop {\sum}\nolimits_{k \in \left[ { - \pi ,\pi } \right]} {\frac{1}{2}\left[ {{{{\mathrm{I}}}} + {{{\mathcal{P}}}}\left( k \right)\left( {{{{\mathbf{n}}}}_i \cdot {{{\mathbf{n}}}}_{{{\mathcal{H}}}}} \right){{{\mathbf{n}}}}_{{{\mathcal{H}}}} \cdot \vec \sigma } \right]}$, where I is the 2 × 2 identity matrix, ${{{\mathcal{P}}}}\left( k \right)$ represents the initial probability distribution in momentum space, and **n**_*i*_ denotes the initial Bloch vector. Moreover, the steady state can be obtained by tracing out bath ${{{\mathcal{B}}}}$ from the “diagonal ensemble”^6^ or be directly calculated by averaging the long-time dynamics of the spin subsystem^43^ (for details, see Supplementary Section A). Since the spin subsystem reaches a steady state, all the local observables on the spin are obviously equilibrated. Generally, the steady state $\rho _{{{{\mathrm{st}}}}}$ explicitly depends on ${{{\mathcal{P}}}}\left( k \right)$ and **n**_*i*_; thus, it remains the details of the system’s initial conditions. Without further insights, we cannot predict the steady state through the system’s conserved quantities, which are supposed to be substantially fewer than the number of degrees of freedom of the whole system.

### Failure of the ETH

From the perspective of the “universality” of thermalization, the prediction of the steady state of the spin subsystem gets a loss of effectiveness, which can be observed from the breakdown of ETH in QW. Conventionally, the ETH states that each energy eigenstate can be thermal, that is, any eigenstate with energy *E*_*α*_ locally equals the uniformly distributed ME, which can be expressed as^53^

1 $${{{\mathrm{Tr}}}}_{{{\mathcal{B}}}}\left[ {\left| {E_\alpha } \right\rangle \left\langle {E_\alpha } \right|} \right] = {{{\mathrm{Tr}}}}_{{{\mathcal{B}}}}\left[ {\frac{{{{{\mathrm{I}}}}_{{{{\mathscr{H}}}}_{\delta E_{{{{\mathrm{ME}}}}}}}}}{{\dim \left( {{{{\mathscr{H}}}}_{\delta E_{{{{\mathrm{ME}}}}}}} \right)}}} \right]$$

where ${{{\mathrm{Tr}}}}_{{{\mathcal{B}}}}$ takes the trace over the bath, $\dim \left( {{{{\mathscr{H}}}}_{\delta E_{{{{\mathrm{ME}}}}}}} \right)$ denotes the dimension of the Hilbert subspace ${{{\mathscr{H}}}}_{\delta E_{{{{\mathrm{ME}}}}}}$ spanned by the eigenstates whose energy belongs to the energy window $[E_\alpha - \delta E_{{{{\mathrm{ME}}}}},E_\alpha + \delta E_{{{{\mathrm{ME}}}}}]$, and ${{{\mathrm{I}}}}_{{{{\mathscr{H}}}}_{\delta E_{{{{\mathrm{ME}}}}}}}$ is the identity matrix defined on this subspace. Additionally, the energy window whose size is determined by $\delta E_{{{{\mathrm{ME}}}}}$ should be macroscopically small to make energy fluctuation small but microscopically large to cover enough energy levels. The ETH guarantees that any superposition state of the energy eigenstates with similar energy can give the same local reduced density matrix, recovering the “universality” of the thermalization. While the ETH is sufficient for understanding the thermalization in non-integrable quantum systems, the existence of the non-trivial conserved quantities and energy degeneracy breaks the validity of this hypothesis down in integrable systems^14^. For the case of the QW model, since the energy eigenstates with opposite momentum ±*k* have the same energy, without loss of generality, the system’s eigenstate in the degenerate subspace with energy *E*_0_ can be represented as $\left| {E_0} \right\rangle = a_ + \left| {{{{\mathbf{n}}}}_{{{\mathcal{H}}}}(k_0)} \right\rangle \otimes \left| {k_0} \right\rangle + a_ - \left| {{{{\mathbf{n}}}}_{{{\mathcal{H}}}}( - k_0)} \right\rangle \otimes \left| { - k_0} \right\rangle$, where *a*_±_ are the normalized complex amplitudes. However, as shown in Supplementary Section C, the reduced density matrix of this eigenstate explicitly depends on the details of the amplitudes *a*_±_, indicating a failure of the ETH.

### Generalized thermalization in QW

Even though the spin subsystem cannot relax to a thermal state, we show herein that the steady state $\rho _{{{{\mathrm{st}}}}}$ can still be predicted by the generalized micro-canonical ensemble (GME)^28,54^ and exhibits generalized thermalization (see the Supplementary Section D for details). To understand this generalized thermalization^28^, the ETH should be extended by generalizing the system’s energy eigenstates to the mutual eigenstates of the Hamiltonian ${{{\mathcal{H}}}}_{{{{\mathrm{eff}}}}}$ and the additional non-trivial conserved quantity -- momentum ${{{\mathcal{K}}}} = \mathop {\sum}\nolimits_{k \in [ - \pi ,\pi ]} {k\left| k \right\rangle \left\langle k \right|}$ with *k* in the first Brillouin zone. According to the GETH, each mutual eigenstate $\left| {E_\alpha ,k_\beta } \right\rangle$ with the energy *E*_*α*_ and momentum *k*_*β*_ can be generalized thermal, that is, the mutual eigenstate with energy *E*_*α*_ and momentum *k*_*β*_ locally equals the GME state, which can be expressed as^54^

2 $${{{\mathrm{Tr}}}}_{{{\mathcal{B}}}}\left[ {\left| {E_\alpha ,k_\beta } \right\rangle \left\langle {E_\alpha ,k_\beta } \right|} \right] = {{{\mathrm{Tr}}}}_{{{\mathcal{B}}}}\left[ {\frac{{{\mathop{\rm I}\nolimits} _{{{{\mathscr{H}}}}_{\{ \delta E_{{{{\mathrm{GME}}}}},\delta k_{{{{\mathrm{GME}}}}}\} }}}}{{\dim \left( {{{{\mathscr{H}}}}_{\{ \delta E_{{{{\mathrm{GME}}}}},\delta k_{{{{\mathrm{GME}}}}}\} }} \right)}}} \right]$$

where ${{{\mathscr{H}}}}_{\{ \delta E_{{{{\mathrm{GME}}}}},\delta k_{{{{\mathrm{GME}}}}}\} }$ is the Hilbert subspace, which is spanned by the eigenstates whose energy and momentum are within the energy window $[E_\alpha - \delta E_{{{{\mathrm{GME}}}}},E_\alpha + \delta E_{{{{\mathrm{GME}}}}}]$ and momentum window $[k_\beta - \delta k_{{{{\mathrm{GME}}}}},k_\beta + \delta k_{{{{\mathrm{GME}}}}}]$ respectively, and ${\mathop{\rm I}\nolimits} _{{{{\mathscr{H}}}}_{\{ \delta E_{{{{\mathrm{GME}}}}},\delta k_{{{{\mathrm{GME}}}}}\} }}$ is the identity matrix in this subspace. Moreover, these windows of conserved quantities should also be macroscopically small but microscopically large. For convenience, we label the reduced density matrix of the spin subsystem on the right-hand side of Eq. (2) as $\rho _{{{{\mathrm{GETH}}}}}(E_\alpha ,k_\beta )$. Based on the GETH, any superposition state of the mutual eigenstates with similar conserved quantities can locally relax to the same reduced state, which is independent of the details of the initial state. Consequently, the “universality” of the steady state $\rho _{{{{\mathrm{st}}}}}$ can be recovered. Particularly, the degeneracy of the energy eigenstate causing the failure of the ETH disappears in the mutual eigenstate under GETH. Herein, in a photonic discrete-time QW, we will experimentally verify the GETH and generalized thermalization. As an isolated integrable quantum system, its energy level has two-fold degeneracy, and there exist additional non-trivial conserved quantities.

## Experimental results

### Observation of the spin subsystem equilibration

The photonic QW was implemented based on the time-multiplexing protocol depicted in Fig. 2b. We took the two pseudo-spin states $\left| {H(V)} \right\rangle \leftrightarrow \left| { \uparrow ( \downarrow )} \right\rangle$ of a single photon to composite the spin (coin) subsystem, where $\left| {H(V)} \right\rangle$ denotes the horizontal (vertical) polarization, and adopted its arriving time to engineer the lattice subsystem. Initially, the walker is localized at the original site (*x* = 0); after an *n*-step walk, it will occupy the sites $\left| x \right| \le n$ and $P(\left| x \right| > n) = 0$.

As discussed above, to verify the validity of the GETH, an essential and quite challenging step is to initialize the system to the superposition states of the mutual eigenstates of two conserved quantities $\{ {{{\mathcal{H}}}},{{{\mathcal{K}}}}\}$ within small windows. As illustrated in Fig. 2b and detailedly described in the “Methods”, we initialized the system in a product state by applying the quantum state engineering technique, which reads: $\left| {{\Psi}(0)} \right\rangle = \left| {\psi _0} \right\rangle _{{{\mathcal{S}}}} \otimes \left| {\psi _0} \right\rangle _{{{\mathcal{B}}}} = \left[ {a_{k_0}\left| {{{{\mathbf{n}}}}_{k_0}^{{{\mathrm{u}}}}} \right\rangle + b_{k_0}\left| {{{{\mathbf{n}}}}_{k_0}^{{{\mathrm{d}}}}} \right\rangle } \right] \otimes \mathop {\sum}\nolimits_x {e^{ixk_0}\sqrt {P(x)} \left| x \right\rangle }$. The $\left| {{{{\mathbf{n}}}}_{k_0}^{{{\mathrm{u}}}(\mathrm{d})}} \right\rangle$ denotes the eigenstates of ${{{\mathbf{n}}}}_{{{\mathcal{H}}}}(k_0) \cdot \vec \sigma$ with the corresponding eigenvalue ±1, $a_{k_0}$ and $b_{k_0}$ are the complex coefficients satisfying $\left| {a_{k_0}} \right|^2 + \left| {b_{k_0}} \right|^2 = 1$, $P(x)$ is a Gaussian distribution peaked around the original site $\left| {x = 0} \right\rangle$ and with a standard deviation Δ_*x*_, and $e^{ixk_0}$ gives the local phase of each site. Consequently, the initial state in the momentum space takes the form of a Gaussian wavepacket peaked around *k*_0_ and with a standard deviation ${\Delta}_k = 1/(2{\Delta}_x)$. In addition, when ${\Delta}_k \to 0$ and $b_{k_0} = 0$ ($a_{k_0} = 0$), the initial state can obviously approach the upper (lower) band mutual eigenstate $\left| {{{{\mathbf{n}}}}_{k_0}^{{{{\mathrm{u(d)}}}}}} \right\rangle \otimes \left| {k_0} \right\rangle$ whose energy is $E_{k_0}^{{{{\mathrm{u(d)}}}}} = \pm E(k)$ and momentum is *k*_0_, satisfying the condition in the GETH. Benefiting from the quantum state engineering technique, we can verify the GETH by asymptotically reducing Δ_*k*_. It is noteworthy that the Δ_*k*_ in our experiments also plays a similar role as the half-width of the conserved quantity window $\delta k_{{{{\mathrm{GME}}}}}$ in Eq. (2).

**Fig. 3:**
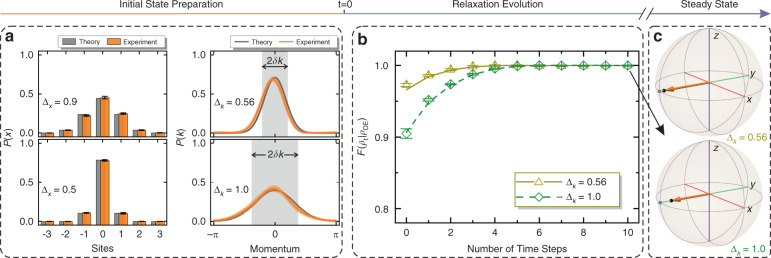
Observing the spin subsystem equilibration. **a** Measured initial Gaussian distributions (orange bars in the left two panels) $P(x)$ with corresponding ${\Delta}_x = 0.9$ and ${\Delta}_x = 0.5$ in the lattice space, and the associated distributions (solid orange lines in the right two panels) ${{{\mathcal{P}}}}(k)$ with corresponding ${\Delta}_k = 0.56$ and ${\Delta}_k = 1.0$ in momentum space. The gray bars and solid gray lines represent the theoretical simulations, and the gray regions denote the width ($2\delta k = 2\sqrt {2\ln 2} {\Delta}_k$) of the distributions. **b** Fidelity ${{{\mathcal{F}}}}(\bar \rho _t|\rho _{{{{\mathrm{DE}}}}})$ (yellow and green symbols) between the measured time-averaged coin state and diagonal ensemble prediction at each time step starting from the prepared two-bath states coupling to the coin state $\left| { \downarrow _y} \right\rangle$. The lines (solid yellow and dashed green) give the theoretical simulations. **c** Bloch sphere representations of the initial coin states (gray points), ten-step-averaged coin states (black points), and diagonal ensemble predictions (pointed by the orange arrows). All the error bars represent the statistical errors estimated by applying the Monte Carlo simulations

In the left two panels of Fig. 3a, we plot the experimentally measured distributions *P*(*x*) of the system’s two initial states, both of which occupy seven sites and have a Gaussian fitting with ${\Delta}_x = 0.9$ and ${\Delta}_x = 0.5$, respectively. Besides, the global wavefunction can also be reconstructed using an interferometric measurement approach^40^. Thus, by applying a Fourier transform, we can further obtain the associated wavefunction in the momentum space and its probability distribution ${{{\mathcal{P}}}}(k)$ centered at *k*_0_ = 0, which are shown in the right two panels. After preparing the bath state, the coin state of interest can further be initialized in $|{\psi _0} \rangle _{{{\mathcal{S}}}} = |{\downarrow _y}\rangle$.

Relaxing the spin subsystem of interest, which is coupled to a bath (discrete infinite lattice), can then be investigated after the proper initialization. The dynamical evolution of the whole system is chosen to be governed by an effective Hamiltonian ${{{\mathcal{H}}}}_{{{{\mathrm{eff}}}}}(7.9\pi /9,8\pi /9)$. The spin–orbit coupling results in the mixture of the spin subsystem, whose time-averaged state $\bar \rho _t$ finally relaxes to a steady state and is in the vicinity of it in most of the time steps, indicating the equilibration of the subsystem^51,52^. As depicted in Fig. 3b, we presented the measured fidelity at different time steps, which is defined as ${{{\mathcal{F}}}}\left( {\bar \rho _t|\rho _{{{{\mathrm{DE}}}}}} \right) = \left[ {{{{\mathrm{Tr}}}}\sqrt {\sqrt {\rho _{{{{\mathrm{DE}}}}}} \bar \rho _t\sqrt {\rho _{{{{\mathrm{DE}}}}}} } } \right]^2$ (where $\rho _{{{{\mathrm{DE}}}}}$ denotes the state obtained by tracing the bath from the “diagonal ensemble” of the mutual eigenstates) and gives the degree of the overlap between the two states. After a relaxation of a six-step walk, the measured fidelity approaches 1 and can maintain this maximal overlap in the subsequent time steps, showing that the spin subsystem can equilibrate to the prediction of a diagonal ensemble. Actually, the steady state of any initial state (arbitrary Δ_*k*_ here) can always be predicted by the diagonal ensemble, which depends on the details of the initial conditions when the GETH is not incorporated. We further plot the ten-step-averaged coin states $\bar \rho _{10}$ (represented by the black points) and the diagonal ensemble predictions (pointed by the orange arrows) in Fig. 3c. Their congruence in the Bloch sphere gives an experimental equivalence between the steady state obtained by the time average and diagonal ensemble prediction. The equilibrium time scale of the spin subsystem *T*_*eq*_ is dependent on the local observable of interest, initial state, and Hamiltonian parameters^55^ (for details, see Supplementary Section B). To balance the clearness of the experimental phenomena and the experimental challenge for maintaining coherence, here we choose the proper set of $\{ \theta _1,\theta _2\}$ with *T*_*eq*_ being small and thus a 10-step QW is enough for the spin subsystem equilibrium.

### Verification of the generalized ETH

**Fig. 4:**
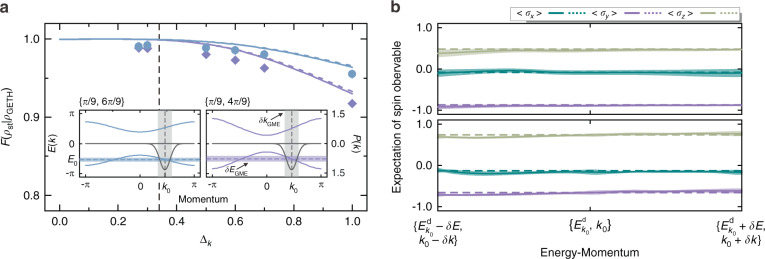
Verifying the generalized eigenstate thermalization hypothesis. **a** Fidelity ${{{\mathcal{F}}}}(\rho _{{{{\mathrm{st}}}}}|\rho _{{{{\mathrm{GETH}}}}})$ between the measured steady state $\rho _{{{{\mathrm{st}}}}}$ and GME prediction $\rho _{{{{\mathrm{GETH}}}}}$ against the standard deviation Δ_*k*_ for a walk with (blue circles) ${{{\mathcal{H}}}}_{{{{\mathrm{eff}}}}}(\pi /9,6\pi /9)$ and (purple diamonds) ${{{\mathcal{H}}}}_{{{{\mathrm{eff}}}}}(\pi /9,4\pi /9)$. The colored dashed and solid lines represent the theoretical simulations for the steady state estimated by $\bar \rho _{t = 10}$ and $\bar \rho _{t \to \infty }$, respectively. The dashed black line represents the upper bound of the initial Δ_*k*_; below this line, the fidelity always approaches 1. The insets illustrate the effective band structures (colored solid lines with left-hand scale) and occupations of the initial state (with ${\Delta}_k = 0.3$ as an example) on the two bands (gray curves with right-hand scale). The expectation of the energy and momentum of the initial state is *E*_0_ and *k*_0_, respectively. In the insets, the vertical gray regions and horizontal colored regions show the chosen momentum window with $\delta k = 0.4$ and associated energy window. **b** Measured expectations of the three spin observables of the reconstructed spinor eigenvectors ${{{\mathbf{n}}}}_{{{\mathcal{H}}}}(k)$ within the chosen energy-momentum window. The upper and lower panels show the results for ${{{\mathcal{H}}}}_{{{{\mathrm{eff}}}}}(\pi /9,6\pi /9)$ and ${{{\mathcal{H}}}}_{{{{\mathrm{eff}}}}}(\pi /9,4\pi /9)$, respectively. The colored dashed lines give the theoretical results for $\rho _{{{{\mathrm{GETH}}}}}(E_{k_0}^{{{\mathrm{d}}}},k_0)$, and the translucent shadings illustrate the statistical errors

To verify the GETH in our integrable system, the initial state of the system takes the form of a Gaussian wavepacket peaked around $k_0 = 6\pi /13$ with a width Δ_*k*_, whose coin state is set to be $\left| {{{{\mathbf{n}}}}_{k_0}^{{{\mathrm{d}}}}} \right\rangle$. The following spin subsystem relaxation, which was effectively governed by two Hamiltonians with $\{ \theta _1,\theta _2\}$ equaling $\{ \pi /9,6\pi /9\}$ and $\{ \pi /9,\,4\pi /9\}$, was experimentally observed. We compared its steady state $\rho _{{{{\mathrm{st}}}}}$ that was experimentally estimated by $\bar \rho _{10}$ with the GME prediction $\rho _{{{{\mathrm{GETH}}}}}(E_{k_0}^{{{\mathrm{d}}}},k_0)$, and the measured fidelity ${{{\mathcal{F}}}}(\rho _{{{{\mathrm{st}}}}}|\rho _{{{{\mathrm{GETH}}}}})$ as a function of initial Δ_*k*_ is plotted as points in Fig. 4a. The GME is chosen within a momentum window centered at *k*_0_ with a half-width $\delta k_{{{{\mathrm{GME}}}}} = 0.4$ (the associated energy window half-width $\delta E_{{{{\mathrm{GME}}}}}$ can be further determined based on the function *E*(*k*)), as exhibited in the inset. Herein, we suppose that the lattice space is infinite and $P(\left| x \right| > n) = 0$ in real space, where *n* is the total number of steps taken by the walker. Consequently, within a proper momentum window, the macroscopically small but microscopically large condition can always be satisfied.

Besides, by choosing a different Δ_*k*_, that is, the initial state corresponding to different superposition states of the mutual eigenstates within the window *δk*, we observed that the ${{{\mathcal{F}}}}(\rho _{{{{\mathrm{st}}}}}|\rho _{{{{\mathrm{GETH}}}}})$ approximates 1 and gets stable. This occurs when the initial Δ_*k*_ is smaller than a value ${\Delta}_k^{\max }$, which is guided by the vertical dashed line and can be theoretically estimated based on the maximal value of the momentum standard deviation of the GME ${\Delta}k_{{{{\mathrm{GME}}}}}$, as shown in Supplementary Section D. Thus, the results directly indicate the “university” of the local steady state, that is, it is independent of the detailed form of the superposition state and only depends on the conserved quantities $\{ E_{k_0}^{{{\mathrm{d}}}},k_0\}$ when Δ_*k*_ is small enough ($< 0.340$ in this situation). In addition, ${{{\mathcal{F}}}}(\rho _{{{{\mathrm{st}}}}}|\rho _{{{{\mathrm{GETH}}}}})$ approaches 1 when ${\Delta}_k \to 0$ gives a direct verification of the GETH. For large Δ_*k*_, the lower fidelity indicates that the steady state differs from the prediction of the GME, signifying its failure.

Further, the expectation of a local observable almost does not fluctuate between the mutual eigenstates that have a similar value of the conserved quantities with each other^6,27^ is another verification of the validity of the GETH. In our system, the spinor eigenvectors ${{{\mathbf{n}}}}_{{{\mathcal{H}}}}(k)$ can be experimentally obtained through the dynamics of spin state^40^, from which we can further get the expectations of the spin observables along with the energy-momentum of the system. In Fig. 4b, we plot the expectations of $\{ \sigma _x,\sigma _y,\sigma _z\}$ of ${{{\mathbf{n}}}}_{{{\mathcal{H}}}}(k)$ within the energy-momentum window. Note that both the momentum and energy windows are necessary to the tiny fluctuation of the expectations. Thus, if we only limit one conserved quantity (such as energy in ETH), and have no information about the other quantity (momentum), then ${{{\mathbf{n}}}}_{{{\mathcal{H}}}}(k)$ have two different choices (±*k*) because of its degeneracy. Therefore, the expectations of observables can have large fluctuation. However, using both of the two conserved quantities, the tiny fluctuation of the expectations can be guaranteed, as shown in Fig. 4b.

### Observation of the generalized thermalization

Besides the superposition state of mutual eigenstates with conserved quantities in a small connected window, the GETH can also be employed to understand the generalized thermalization of the superposition state of mutual eigenstates in two separated windows^28^ (for details, see Supplementary Section E).

**Fig. 5:**
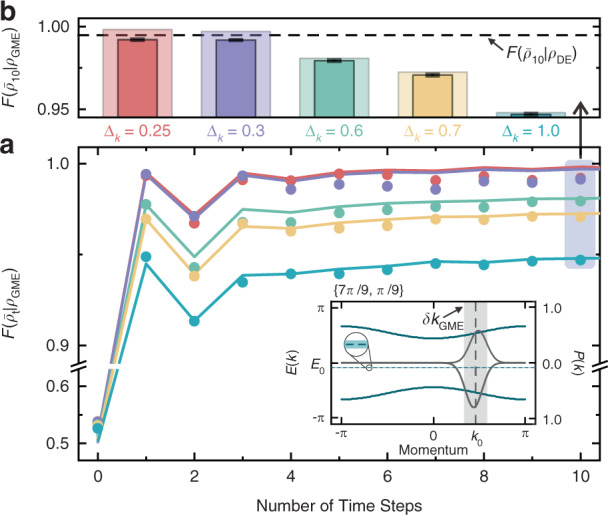
Observing the generalized thermalization. **a** Fidelity (dots) between the measured time-averaged coin states $\bar \rho _t$ and GME prediction $\rho _{{{{\mathrm{GME}}}}}$ against the time steps, and **b** their values (opaque bars) after a ten-step walk. The initial Gaussian states have five different widths Δ*k* but the same coin state $\left| { \downarrow _y} \right\rangle$, and then evolve governed by ${{{\mathcal{H}}}}_{{{{\mathrm{eff}}}}}(7\pi /9,\pi /9)$. The colored solid lines in (**a**) and transparent bars in (**b**) represent the theoretical simulations. The inset in (**a**) shows the effective band structures (solid green lines with left-hand scale), the occupations of the two bands for one initial state with ${\Delta}_k = 0.3$ as an example (gray curves with right-hand scale). The expected energy and momentum of the initial state is *E*_0_ and *k*_0_, respectively. After expanding the initial state, the shift of the mean momentum of each band occupation relative to the *k*_0_ takes the following form: $\mathop {\sum}\nolimits_{\delta k} {{{{\mathcal{P}}}}(k){{{\mathcal{O}}}}(k - k_0)}$, where ${{{\mathcal{O}}}}$ denotes the order of the function. Moreover, the vertical gray and horizontal green regions depict the chosen momentum window with $\delta k_{{{{\mathrm{GME}}}}} = 0.4$ and associated energy window, respectively. The dashed line in (**b**) indicates the lower bound (99.42%) of the measured ${{{\mathcal{F}}}}(\bar \rho _{10}|\rho _{{{{\mathrm{DE}}}}})$

As an example (for another example, see Supplementary Section E), we investigated the relaxation of the coherent superposition of the mutual eigenstates given by $\left| {{\Psi}(0)} \right\rangle = \mathop {\sum}\nolimits_{|k - k_0| < \delta k} {\left[ {a_k\left| {{{{\mathbf{n}}}}_k^{{{\mathrm{u}}}}} \right\rangle + b_k\left| {{{{\mathbf{n}}}}_k^{{{\mathrm{d}}}}} \right\rangle } \right] \otimes \sqrt {{{{\mathcal{P}}}}(k)} \left| k \right\rangle }$. Experimentally, the initial state remains a Gaussian wavepacket peaked around $k_0 = 6\pi /13$ with different standard deviations Δ_*k*_ and the coin state $\left| {\psi _0} \right\rangle _{{{\mathcal{S}}}}$ is prepared in $\left| { \downarrow _y} \right\rangle$, as shown in the inset of Fig. 5a. Moreover, we observed the following evolution, which is governed by an effective Hamiltonian ${{{\mathcal{H}}}}_{{{{\mathrm{eff}}}}}(7\pi /9,\pi /9)$, and plot the measured fidelity ${{{\mathcal{F}}}}(\bar \rho _t|\rho _{{{{\mathrm{GME}}}}})$ for five initial states with different Δ_*k*_ in Fig. 5a. Note that $\rho _{{{{\mathrm{GME}}}}}$ denotes the local reduced state obtained by tracing the bath from the GME, whose energy-momentum window is shown in the inset.

Besides, the experimental results show that the measured fidelity reaches a steady value, indicating the steady state of the spin subsystem. The steady state should match $\rho _{{{{\mathrm{DE}}}}}$ for any initial state, and all the experimentally measured values of fidelity between them are greater than $99.42\%$ represented by the dashed line in Fig. 5b.

Similar to the situation in a connected window, when the initial Δ_*k*_ is large (for example, ${\Delta}_k = 1.0$, 0.7, and 0.6) where the GME prediction fails as experimentally demonstrated before, the steady states estimated by $\bar \rho _{10}$ cannot be properly predicted by $\rho _{{{{\mathrm{GME}}}}}$, as shown in the Fig. 5b. However, when Δ_*k*_ is sufficiently small (${\Delta}_k\, < \,{\Delta}_k^{\max } = 0.340$), such as ${\Delta}_k = 0.3$ and 0.25, due to the validity of the GETH, the steady states agree with the prediction of the GME. Thus, the validity of the GETH can also render the diagonal ensemble and the GME to be locally equivalent in this extended situation.

## Discussion

Herein, in an isolated integrable quantum system, we experimentally observed that the relaxation of the spin subsystem of interest always agrees with the prediction of the “diagonal ensemble” of the mutual eigenstates. Importantly, by applying the quantum state engineering and eigenvectors reconstruction techniques, the GETH has been verified for the first time in an asymptotic method by comparing the steady spin state, starting from the initial states with different distribution widths, with the GME prediction. We further demonstrated that the combination of the GETH and the diagonal ensemble can be used to understand the generalized thermalization in integrable systems. Although these results in our work utilize the quantum coherence involving the two inner degrees of freedom of the photons, it doesn’t rely on whether the system involves the higher-order coherence and can then be used to promote understanding of the quantum thermalization in a many-body version. Moreover, our novel setup with low transmission loss also has the potential to implement protocols involving high-order coherence. We believe our experimental findings as well as the creative platform could enable the understanding of the basic theory of quantum statistical mechanics in a general quantum system.

## Materials and methods

### Discrete-time QW

The unitary operator for a complete step of QW is given by $U = {{{\mathcal{T}}}}_ \uparrow R_2{{{\mathcal{T}}}}_ \uparrow R_1$, where the coin-tossing $R_{1(2)}$ acting on coin space can be any operator from the SU(2) group, and ${{{\mathcal{T}}}}_{ \uparrow ( \downarrow )} = \mathop {\sum}\nolimits_x {\left( {\left| {x \pm 1} \right\rangle \left\langle x \right| \otimes \left| { \uparrow ( \downarrow )} \right\rangle \left\langle { \uparrow ( \downarrow )} \right| + \left| x \right\rangle \left\langle x \right| \otimes \left| { \downarrow ( \uparrow )} \right\rangle \left\langle { \downarrow ( \uparrow )} \right|} \right)}$ denote the spin-dependent hopping operators. The exact form of the effective Hamiltonian ${{{\mathcal{H}}}}_{{{{\mathrm{eff}}}}}$ depends on the details of the coin-tossing. Given the rotations along the y-axis, i.e., $R_{1(2)} = R_y(\theta _{1(2)})$ with $\theta _{1(2)}$ representing the rotation angles, we can get the momentum-dependent energy $\cos [E(k)] = \cos (\theta _1/2)\cos (\theta _2/2)\cos k - \sin (\theta _1/2)sin(\theta _2/2)$ and the corresponding spinor eigenvector^49^

3 $$\left\{ {\begin{array}{*{20}{c}} {n_{{{\mathcal{H}}}}^x(k) = \frac{{\sin (\theta _1/2)\cos (\theta _2/2)\sin k}}{{\sin [E(k)]}}} \\ {n_{{{\mathcal{H}}}}^y(k) = \frac{{\sin (\theta _1/2)\cos (\theta _2/2)\cos k + \cos (\theta _1/2)\sin (\theta _2/2)}}{{\sin [E(k)]}}} \\ {n_{{{\mathcal{H}}}}^z(k) = \frac{{ - \cos (\theta _1/2)\cos (\theta _2/2)\sin k}}{{\sin [E(k)]}}} \end{array}} \right.$$

### Heralded single-photon source

The pulsed laser emitting from a Ti:Sapphire source (Mira 900) has a central wavelength at 800 nm, a repetition rate of 76 MHz, and a pulse duration of 150 fs, and it is focused by applying L1 to pump a piece of nonlinear crystal BBO1. Moreover, the type-I second harmonic occurring in BBO1 then generates the frequency-doubled ultraviolet laser with a central wavelength of 400 nm and an average power of 150 mW, which is horizontally polarized and focused to pump the second crystal BBO2. The type-II beam-like SPDC that occurs in BBO2 generates correlated photon pairs, where the signal and idler photons are centered at 780 and 821 nm, respectively. The photon pairs are collimated using L4 with a focal length f = 150 mm, and the signal photons heralded by the idler photons are adopted as the walker and guided to the QW module.

### Photonic time-multiplexing implementation

QW has been implemented in various physical systems^56^. Among them, linear optics plays a pivotal role in implementing QW, where the spatial^57^, temporal^47^, and orbital angular momentum^58^ degrees of freedom of photons have been utilized. Previous time-multiplexing configuration employing fiber loop is very compact and enables the realization of large-scale QW. However, the existence of an unavoidable high photon loss necessitates the use of an attenuated coherent laser. In our time-multiplexing implementation, the spin–orbit coupling was realized using the birefringent calcite crystal collinearly cut, whose length is designed to be 8.98 mm yielding a time shift of 5 ps between the two orthogonal polarizations. Thus, a single-photon pulse train having an equal interval of 5 ps comprises the lattice space. Moreover, this QW protocol is compact, and without its extra loss, guaranteeing its applicability to single-photon scenarios. A coin-tossing $R \in {{{\mathrm{SU(2)}}}}$ can be achieved by employing the universal single-qubit gate acting on polarization, which is usually a set of wave plates, i.e., QWP-HWP-QWP in sequence. For the case of rotating along the y-axis, the set of wave plates can be reduced to a single HWP.

### Initial state preparation

QW with time-dependent SU(2) coins can be applied to engineer arbitrary high-dimensional quantum state (qudit) over the lattice space^44,45^. To engineer the qudit state, the walker is initially located at the original site ($\left| {x = 0} \right\rangle$) with its coin encoded in $\left| H \right\rangle$, and at the end of the walk, the coin is projected into an unbiased base $\left| + \right\rangle = \frac{1}{{\sqrt 2 }}\left( {\left| H \right\rangle + \left| V \right\rangle } \right)$. Moreover, the set of the time-dependent coin operators are found using a numerical optimization, thereby maximizing the fidelity (as a function of the rotation angles) between the target state and final lattice state after the coin projection. In our implementations, the target state is a Gaussian wavepacket with standard deviation Δ_*x*_ and the local phase of each site $e^{ixk_0}$. By applying a Fourier transform, it can also be described in the associated momentum space as $\left| {\psi _0} \right\rangle _{{{\mathcal{B}}}} = A_0\mathop {\sum}\nolimits_{k \in [ - \pi ,\pi ]} {e^{ - (k - k_0)^2/(2{\Delta}_k)^2}} \left| k \right\rangle$ peaked around the momentum *k*_0_, where Δ_*k*_ denotes the standard deviation and *A*_0_ is a normalization constant. The initial coin state $\left| {\psi _0} \right\rangle _{{{\mathcal{S}}}}$ can be arbitrarily set using the wave plates.

## Supplementary information

Supplementary Information for Experimental Verification of Generalized Eigenstate Thermalization Hypothesis in an Integrable System

## Data Availability

Source data are available for this paper. All other data that support the plots within this paper and other findings of this study are available from the corresponding author on reasonable request.
