# Supplementary material for: Experimental verification of generalized eigenstate thermalization hypothesis in an integrable system
Source: Light Sci Appl. 2022 Jun 28;11:194. doi: 10.1038/s41377-022-00887-5 (PMC9239999; doi:10.1038/s41377-022-00887-5)
Supplement: Supplementary file 1 — Supplementary Information for Experimental Verification of Generalized Eigenstate Thermalization Hypothesis in an Integrable System [file 41377_2022_887_MOESM1_ESM.pdf]

Supplementary Information for *Experimental Verification of Generalized Eigenstate Thermalization Hypothesis in an Integrable System*

Qin-Qin Wang,[1,2,3,*] Si-Jing Tao,[1,2,3,*] Wei-Wei Pan,[1,2,3] Zhe Chen,[1,2,3]
Geng Chen,[1,2,3] Kai Sun,[1,2,3] Jin-Shi Xu,[1,2,3] Xiao-Ye Xu,[1,2,3,†]
Yong-Jian Han,[1,2,3,‡] Chuan-Feng Li,[1,2,3,§] and Guang-Can Guo[1,2,3]

[1] *CAS Key Laboratory of Quantum Information, University of Science and Technology of China, Hefei 230026, China*

[2] *CAS Center for Excellence in Quantum Information and Quantum Physics, University of Science and Technology of China, Hefei 230026, China*

[3] *Hefei National Laboratory, University of Science and Technology of China, Hefei 230088, China*

(Dated: June 1, 2022)

A. EQUILIBRATION DYNAMICS OF THE SPIN-ORBIT COUPLING SYSTEM

The isolated quantum system evolves unitarily, and it seems that equilibration cannot occur in such system. However, many investigations have shown theoretically and experimentally that a part of the isolated quantum system (the rest part served as a bath) can equilibrate when the effective dimension of the initial state is large enough [1–5]. For the spin-orbit coupling (SOC) quantum system adopted in our experiment, its Hamiltonian takes the form $\mathcal{H}=\sum_k E(k)(\mathbf{n}_{\mathcal{H}}(k)\cdot\vec{\sigma})\otimes|k\rangle\langle k|$, where $E(k)$ is the energy, $\mathbf{n}_{\mathcal{H}}(k)=(n^x_{\mathcal{H}}(k),n^y_{\mathcal{H}}(k),n^z_{\mathcal{H}}(k))$ denotes the axis for the spinor eigenstates at each momentum k, and $\vec{\sigma}=(\sigma_x,\sigma_y,\sigma_z)$ is the Pauli matrix. For a split-step quantum walk (SSQW) [6], we can get the exact form of the (quasi-)energy $\cos[E(k)]=\cos(\theta_1/2)\cos(\theta_2/2)\cos k-\sin(\theta_1/2)\sin(\theta_2/2)$ and the corresponding spinor eigenvector

$$\begin{cases} n^x_{\mathcal{H}}(k)=\dfrac{\sin(\theta_1/2)\cos(\theta_2/2)\sin k}{\sin[E(k)]}\\ n^y_{\mathcal{H}}(k)=\dfrac{\sin(\theta_1/2)\cos(\theta_2/2)\cos k+\cos(\theta_1/2)\sin(\theta_2/2)}{\sin[E(k)]}\\ n^z_{\mathcal{H}}(k)=\dfrac{-\cos(\theta_1/2)\cos(\theta_2/2)\sin k}{\sin[E(k)]}, \end{cases} \tag{S1}$$

where θ_1 and θ_2 are the system's control parameters.

Without loss of generality, we can set the initial state as

$$|\Psi(0)\rangle=\sum\nolimits_k (a_k|\mathbf{n}^u_k\rangle+b_k|\mathbf{n}^d_k\rangle)\otimes\sqrt{\mathcal{P}(k)}|k\rangle, \tag{S2}$$

where a_k and b_k are the normalized complex amplitudes satisfying $|a_k|^2+|b_k|^2=1$, $\mathcal{P}(k)$ is the initial probability distribution in momentum space, $|\mathbf{n}^{u(d)}_k\rangle$ denotes the spinor eigenstates in the upper (lower) energy band with the momentum k, and $|k\rangle$ is eigenstate of the momentum operator. At any given time t, the time-evolving density matrix of the spin subsystem can be written as [7]

$$\begin{aligned}\rho(t)=&\frac{1}{2}\{\mathrm{I}+\sum\nolimits_k \mathcal{P}(k)[(\mathbf{n}_i(k)\cdot\mathbf{n}_{\mathcal{H}}(k))\mathbf{n}_{\mathcal{H}}(k)\cdot\vec{\sigma}\\ &-\cos[2E(k)t](\mathbf{n}_i(k)\cdot\mathbf{n}_{\mathcal{H}}(k))\mathbf{n}_{\mathcal{H}}(k)\cdot\vec{\sigma}+\cos[2E(k)t]\mathbf{n}_i(k)\cdot\vec{\sigma}\\ &+\sin[2E(k)t](\mathbf{n}_{\mathcal{H}}(k)\times\mathbf{n}_i(k))\cdot\vec{\sigma}]\},\end{aligned} \tag{S3}$$

* These authors contributed equally.

† xuxiaoye@ustc.edu.cn

‡ smhan@ustc.edu.cn

§ cfli@ustc.edu.cn

where $\mathbf{n}_i(k)$ denotes the Bloch vector of the initial spin state at each momentum k and satisfies $\mathbf{n}_i(k) \cdot \mathbf{n}_{\mathcal{H}}(k) = |a_k|^2 - |b_k|^2$.

Moreover, the steady state for any initial state can be obtained by using its "diagonal ensemble" [8, 9] as $\rho_{\mathrm{DE}} \equiv \sum_\alpha |c_\alpha|^2 |\alpha\rangle\langle\alpha|$, where $|\alpha\rangle$ is the eigenstate of the Hamiltonian, and $c_\alpha = \langle\alpha|\phi_0\rangle$ is the initial coefficient. Thus, we can predict the steady state of the spin subsystem as

$$\begin{aligned}\rho_{\mathrm{st}} &= \mathrm{Tr}_{\mathcal{B}}[\rho_{\mathrm{DE}}] \qquad \text{(S4)}\\ &= \mathrm{Tr}_{\mathcal{B}}[\sum\nolimits_k (|a_k|^2 |\mathbf{n}_k^u\rangle\langle\mathbf{n}_k^u| + |b_k|^2 |\mathbf{n}_k^d\rangle\langle\mathbf{n}_k^d|) \otimes \mathcal{P}(k)|k\rangle\langle k|]\\ &= \sum\nolimits_k \frac{1}{2}[\mathrm{I} + \mathcal{P}(k)(\mathbf{n}_i(k)\cdot\mathbf{n}_{\mathcal{H}}(k))\mathbf{n}_{\mathcal{H}}(k)\cdot\vec{\sigma}].\end{aligned}$$

Then the dynamics of the reduced density matrix of the spin subsystem can be investigated by comparing the time-evolving state with the steady state ρ_{st}. Herein, we use the trace distance $\mathcal{D}$ to measure the difference between two states as

$$\mathcal{D} = \frac{1}{2}||\rho(t) - \rho_{\mathrm{st}}||_1 \qquad \text{(S5)}$$

$$\begin{aligned}= \Big|\sum\nolimits_k \mathcal{P}(k)\{&\cos[2E(k)t]\mathbf{n}_i(k) - \cos[2E(k)t](\mathbf{n}_i(k)\cdot\mathbf{n}_{\mathcal{H}}(k))\mathbf{n}_{\mathcal{H}}(k)\\ &+ \sin[2E(k)t](\mathbf{n}_{\mathcal{H}}(k)\times\mathbf{n}_i(k))\}\Big|, \qquad \text{(S6)}\end{aligned}$$

where $||\cdot||$ denotes Schatten 1-norm of a matrix, and $|\cdot|$ denotes the norm of a vector. If $\mathcal{P}(k)$ is dense enough in momentum space, such that we can approximately replace the sum by the integral as $\sum_k \to \int dk$, which can be satisfied for the infinite lattice. Moreover, by applying the Riemann-Lebesgue Lemma, $\mathcal{D}$ can vanish in the long-time limit. The SOC system thus relaxes to a steady state that can be predicted by the "diagonal ensemble". It is noteworthy that ρ_{st} can also be directly obtained based on Eq. (S3) through the long-time average of the spin states [2, 5].

B. EQUILIBRATION TIME SCALE OF THE ISOLATED QUANTUM SYSTEM.

In this part, we will determine the the upper bound on the equilibration time scale T_{eq} for the isolated quantum system [10]. Here, we use a normalized trace distance, comparing the time-evolved expected value of an observable A to its equilibrium value, to give a measurement for the distinguishability between the evolved state ρ_t and the long-time average

$\rho_{\rm DE}$

$$\langle \mathcal{D}_A^N(\rho_t, \rho_{\rm DE})\rangle_T \equiv \frac{1}{T}\int_0^T dt \frac{|{\rm Tr}[\rho_t A] - {\rm Tr}[\rho_{\rm DE} A]|^2}{|{\rm Tr}[\rho_0 A] - {\rm Tr}[\rho_{\rm DE} A]|^2}. \tag{S7}$$

Here, the initial density matrix $\rho_0 = \sum_{i,j}\rho_{i,j}|E_i\rangle\langle E_j|$ where $|E_i\rangle$ is the eigenvalue of the Hamiltonian $\mathcal{H}$, and $\rho_{\rm DE} = \sum_i \rho_{i,i}|E_i\rangle\langle E_i|$ is the diagonal ensemble. Then, we have

$$\begin{aligned}\langle \mathcal{D}_A^N(\rho_t, \rho_{\rm DE})\rangle_T &= \frac{1}{T}\int_0^T dt \frac{|\sum_{j\neq k} e^{-{\rm i}(E_j - E_k)t}\rho_{j,k}A_{k,j}|^2}{|\sum_{j\neq k}\rho_{j,k}A_{k,j}|^2} \\ &= \frac{1}{T}\int_0^T dt \frac{|\sum_\alpha e^{-{\rm i}(G_\alpha)t} v_\alpha|^2}{|\sum_\alpha v_\alpha|^2} \\ &= \frac{1}{T}\int_0^T dt \frac{|\sum_{\alpha,\beta} e^{-{\rm i}(G_\alpha - G_\beta)t} v_\alpha v_\beta^*|}{|\sum_\alpha v_\alpha||\sum_\beta v_\beta|} \\ &\leqslant \frac{1}{T}\int_0^T dt \frac{\sum_{\alpha,\beta} e^{-{\rm i}(G_\alpha - G_\beta)t} |v_\alpha||v_\beta^*|}{|\sum_\alpha v_\alpha||\sum_\beta v_\beta|},\end{aligned} \tag{S8}$$

where $\{G_1, G_2, ...\}$ is all energy gaps of the Hamiltonian, $v_\alpha \equiv \rho_{j,k}A_{k,j}$. For convenience of the proof, we define a probability distribution

$$p_\alpha = \frac{1}{\mathcal{N}}\frac{|v_\alpha|}{|\sum_\alpha v_\alpha|}, \tag{S9}$$

where the normalization factor $\mathcal{N} = \frac{|\sum_\alpha v_\alpha|}{\sum_\alpha |v_\alpha|}$. Therefore, we get

$$\langle \mathcal{D}_A^N(\rho_t, \rho_{\rm DE})\rangle_T \leqslant \frac{\mathcal{N}^2}{T}\int_0^T dt \sum_{\alpha,\beta} p_\alpha p_\beta e^{-{\rm i}(G_\alpha - G_\beta)t}. \tag{S10}$$

Moreover, based on the result in [10], we have

$$\begin{aligned}\langle \mathcal{D}_A^N(\rho_t, \rho_{\rm DE})\rangle_T &\leqslant \frac{5\pi\mathcal{N}^2}{4T}\int_0^T dt \sum_{\alpha,\beta} p_\alpha p_\beta e^{-(G_\alpha - G_\beta)t} \\ &\leqslant \pi\mathcal{N}^2(4P_{max}(\frac{1}{T})) \\ &\leqslant \pi\mathcal{N}^2[\frac{4d(\epsilon)}{T} + 4\delta(\epsilon)],\end{aligned} \tag{S11}$$

where ϵ can be any positive real number, $P_{\rm max}(\Delta) \equiv \max_{x_0\in\mathbb{R}}\sum_{\delta:G_\delta\in[x_0,x_0+\Delta]} p_\delta$, $d(\epsilon) \equiv \frac{P_{\rm max}(\epsilon)}{\epsilon}$ and $\delta(\epsilon) \equiv P_{\rm max}(\epsilon)$.

According to the inequality Eq. (S11), the system will eventually be equilibrated with respect to A when the second term on the right-hand side is samll $\mathcal{N}^2\delta(\epsilon) << 1$. And the

system wll equilibrate for the time $T >> T_{\rm eq}$, where the equilibration time scale T_{eq}, in which the decay of distinguishability happens, is then given by

$$T_{\rm eq} \equiv 4\pi \mathcal{N}^2 d(\epsilon). \tag{S12}$$

C. FAILURE OF THE EIGENSTATE THERMALIZATION HYPOTHESIS

It is generally believed that thermalization can occur in the chaotic system; it is broken down in the integrable system. This can be explained by utilizing the validity (failure) of the eigenstate thermalization hypothesis in the chaotic (integrable) system. Eigenstate thermalization hypothesis (ETH) suggests that all the energy eigenstates can be locally equal to the thermal state which is obtained by averaging on an uniformly distributed microcanonical ensemble (ME) [8, 11], i.e.,

$$\mathrm{Tr}_{\mathcal{B}}[|E_\alpha\rangle\langle E_\alpha|] = \mathrm{Tr}_{\mathcal{B}}\left[\frac{\mathrm{I}_{\mathscr{H}_{\delta E_{\rm ME}}}}{\dim(\mathscr{H}_{\delta E_{\rm ME}})}\right]. \tag{S13}$$

$|E_\alpha\rangle$ is the eigenstate with energy E_α, $\mathscr{H}_{\delta E_{\rm ME}}$ is the Hilbert subspace spanned by the eigenstates whose energies are belong to the energy window $[E_\alpha - \delta E_{\rm ME}, E_\alpha + \delta E_{\rm ME}]$, $\dim(\cdot)$ denotes the dimension of a space and $\mathrm{I}_{\mathscr{H}_{\delta E_{\rm ME}}}$ is the identity matrix in this subspace. Moreover, the energy window should be macroscopically small but microscopically large to cover enough energy eigenstates [12]. Based on the ETH, all the energy eigenstates with the similar energy should be locally close to each other. As a result, any superposition of the eigenstates whose energies lie within a small energy window can locally relax to an equivalent state.

However, for the case of SSQW, the energy eigenstate has two-fold degeneracy, i.e., $E(k) = E(-k)$. Considering the scenario that two individual eigenstates with the opposite momentum $\pm k_\alpha$ ($k_\alpha > 0$) have a same energy E_α (see Fig. S1), the reduced density matrices of the two eigenstates are totally different

$$\begin{aligned} &\mathrm{Tr}_{\mathbf{B}}[(|\mathbf{n}^{u(d)}_{k_\alpha}\rangle \otimes |k_\alpha\rangle)(\langle \mathbf{n}^{u(d)}_{k_\alpha}| \otimes \langle |k_\alpha|)] = \frac{1}{2}[\mathrm{I} \pm \mathbf{n}_{\mathcal{H}}(k_\alpha)\cdot\vec{\sigma}] \\ &\neq \\ &\mathrm{Tr}_{\mathbf{B}}[(|\mathbf{n}^{u(d)}_{-k_\alpha}\rangle \otimes |-k_\alpha\rangle)(\langle \mathbf{n}^{u(d)}_{-k_\alpha}| \otimes \langle |-k_\alpha|)] = \frac{1}{2}[\mathrm{I} \pm \mathbf{n}_{\mathcal{H}}(-k_\alpha)\cdot\vec{\sigma}], \end{aligned} \tag{S14}$$

Thus, for a general energy eigenstate $|E_\alpha\rangle = a_+|\mathbf{n}_{\mathcal{H}}(k_\alpha)\rangle \otimes |k_\alpha\rangle + a_-|\mathbf{n}_{\mathcal{H}}(-k_\alpha)\rangle \otimes |-k_\alpha\rangle$, its reduced density matrix highly depends on the complex coefficient $a_\pm$, which indicates the breakdown of the ETH. That is to say, only the information of energy is not enough to determine the steady state of the spin subsystem, and the other nontrivial conserved quantity (i.e., the momentum) is necessary to be introduced to the SSQW.

Consider the initial state in a small energy window $[E_0 - \delta E, E_0 + \delta E]$ as

$$|\Psi(0)\rangle = \sum_{\substack{k>0;\\ |k-k_{E_0}|<\delta k_E}} (e^{i\omega_l(k)}|\mathbf{n}_k^{u(d)}\rangle \otimes \sqrt{\mathcal{P}(k)}|k\rangle + e^{i\omega_r(k)}|\mathbf{n}_{-k}^{u(d)}\rangle \otimes \sqrt{\mathcal{P}(-k)}|-k\rangle). \tag{S15}$$

The $\omega_{l(r)}(k)$ is the phase, and the expected energy of the initial state is E_0. Moreover, the positive momentum window $|k - k_{E_0}| < \delta k_E$ is directly gotten by the chosen energy window (see Fig. S1). According to Eq. (S4), the steady state of the spin system can be written as

$$\begin{aligned}
\rho_{\text{st}} &= \sum_{\substack{k>0;\\ |k-k_{E_0}|<\delta k_E}} \frac{1}{2}[\mathrm{I} + \mathcal{P}(k)(\mathbf{n}_i(k)\cdot\mathbf{n}_{\mathcal{H}}(k))\mathbf{n}_{\mathcal{H}}(k)\cdot\vec{\sigma}] \\
&= \frac{1}{2}[\mathrm{I} \pm \sum_{\substack{k>0;\\ |k-k_{E_0}|<\delta k_{\mathrm{E}}}} \mathcal{P}(k)\mathbf{n}_{\mathcal{H}}(k)\cdot\vec{\sigma} \pm \sum_{\substack{k>0;\\ |k-k_{E_0}|<\delta k_{\mathrm{E}}}} \mathcal{P}(-k)\mathbf{n}_{\mathcal{H}}(-k)\cdot\vec{\sigma}] \qquad \text{(S16)} \\
&\xrightarrow{\delta E \text{ is small}} \frac{1}{2}[\mathrm{I} \pm \tilde{\mathcal{P}}_{\mathrm{R}}\mathbf{n}_{\mathcal{H}}(k_{E_0})\cdot\vec{\sigma} \pm \tilde{\mathcal{P}}_{\mathrm{L}}\mathbf{n}_{\mathcal{H}}(-k_{E_0})\cdot\vec{\sigma}],
\end{aligned}$$

where "$\pm$" represents that the chosen energy eigenstate lies in the upper (lower) energy band, and $\tilde{\mathcal{P}}_{\mathrm{R(L)}} \equiv \sum_{\delta k_{\mathrm{E}}} \mathcal{P}(\pm k)$. On the other hand, based on the ETH, the steady state of the spin subsystem can also be predicted by the micro-canonical ensemble as

$$\begin{aligned}
\rho_{\text{th}} &= \mathrm{Tr}_{\mathcal{B}}\left[\frac{\mathrm{I}_{\mathscr{H}_{\delta E_{\mathrm{ME}}}}}{\dim(\mathscr{H}_{\delta E_{\mathrm{ME}}})}\right] \qquad \text{(S17)} \\
&= \mathrm{Tr}_{\mathcal{B}}[\frac{1}{N_{E_0,\delta E_{\mathrm{ME}}}} \sum_{|E-E_0|<\delta E_{\mathrm{ME}}} (|n_k^{u(d)}\rangle \otimes |k\rangle)(\langle n_k^{u(d)}| \otimes \langle k|)] \\
&= \frac{1}{N_{E_0,\delta E_{\mathrm{ME}}}}\frac{1}{2}[\mathrm{I} \pm \sum_{|k-k_{E_0}|<\delta k^E_{\mathrm{ME}}} \mathbf{n}_{\mathcal{H}}(k)\cdot\vec{\sigma} \pm \sum_{|k-k_{E_0}|<\delta k^E_{\mathrm{ME}}} \mathbf{n}_{\mathcal{H}}(-k)\cdot\vec{\sigma}] \qquad \text{(S18)} \\
&\xrightarrow{\delta E_{\mathrm{ME}} \text{ is small}} \frac{1}{2}[\mathrm{I} \pm \frac{1}{2}\mathbf{n}_{\mathcal{H}}(k_{E_0})\cdot\vec{\sigma} \pm \frac{1}{2}\mathbf{n}_{\mathcal{H}}(-k_{E_0})\cdot\vec{\sigma}],
\end{aligned}$$

where $N_{E_0,\delta E_{\mathrm{ME}}}$ is the number of the eigenstates whose energies located in the energy window $[E_0 - \delta E_{\mathrm{ME}}, E_0 + \delta E_{\mathrm{ME}}]$, and δk^E_{ME} is the half-width of the corresponding momentum window. Obviously, $\rho_{\text{st}} \neq \rho_{\text{th}}$, where ρ_{th} dependents on the energy E_0, and ρ_{st} remains the details of

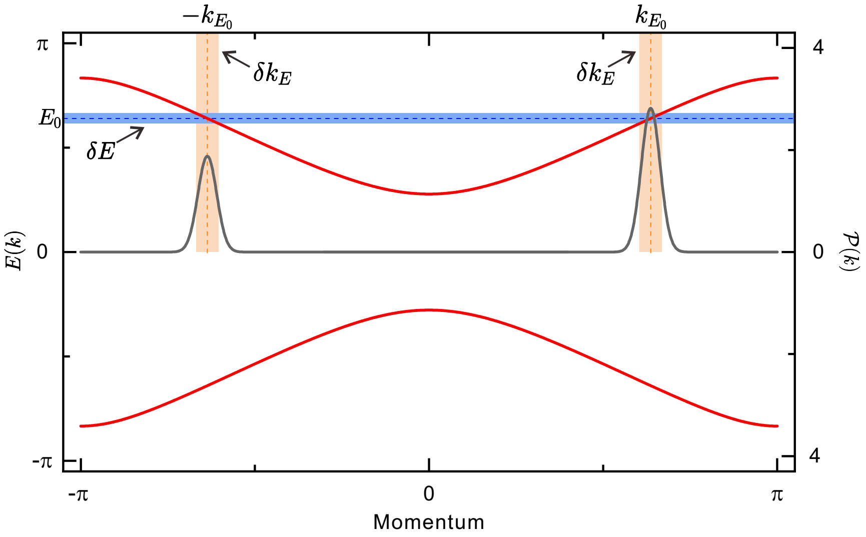

FIG. S1. Diagram of the initial state within a small energy window. The solid red line is the effective energy band structures of the SSQW with $\mathcal{H}_{\text{eff}}(\frac{4\pi}{9}, \frac{\pi}{9})$ [6], and the blue region is a small energy window $[E_0 - \delta E, E_0 + \delta E]$. Because of the two-fold degeneracy of the energy, there are two corresponding momentum windows $[k_{E_0} - \delta k_{\text{E}}, k_{E_0} + \delta k_{\text{E}}]$ and $[-k_{E_0} - \delta k_{\text{E}}, -k_{E_0} + \delta k_{\text{E}}]$, where $k_{E_0} > 0$ satisfying $E(k_{E_0}) = E_0$, and δk_{E} is the half-width of the momentum window, which is determined by δE.

the initial condition $\tilde{\mathcal{P}}_{\text{R(L)}}$. Thus, due to the breakdown of the ETH, the steady state of the spin subsystem cannot be characterized by a thermal state.

D. VALIDITY OF THE GENERALIZED EIGENSTATE THERMALIZATION HYPOTHESIS.

As demonstrated in the previous section, in integrable system such as SSQW, the ETH is broken down; thus, the thermal state fails to characterize the steady state. However, the steady state in integrable system can still be characterized by the generalized micro-canonical ensemble (GME) state [9], and this generalized thermalization is explicable by using the generalized ETH (GETH) [13]. According to the GETH, the mutual eigenstates

of the conserved quantities should be locally equal to the GME state [13, 14], i.e.,

$$\mathrm{Tr}_{\mathcal{B}}\left[|\mathbf{i}_\alpha\rangle\langle\mathbf{i}_\alpha|\right] = \mathrm{Tr}_{\mathcal{B}}\left[\frac{\mathrm{I}_{\mathscr{H}_{\{\delta\mathbf{i}_{\mathrm{GME}}\}}}}{\dim(\mathscr{H}_{\{\delta\mathbf{i}_{\mathrm{GME}}\}})}\right], \tag{S19}$$

where $|\mathbf{i}_\alpha\rangle$ denotes the eigenstate with the values of the conserved quantities $\mathbf{i}_\alpha \equiv \{\mathrm{i}_\alpha^0, \mathrm{i}_\alpha^1, ..., \mathrm{i}_\alpha^N\}$ (in the SSQW, the conserved quantities are the energy and momentum), $\mathscr{H}_{\{\delta\mathbf{i}_{\mathrm{GME}}\}}$ is the Hilbert subspace spanned by the eigenstates whose values of the conserved quantities i^μ ($\mu = 0, 1, 2, ..., N$) lie within the window $[\mathrm{i}_\alpha^\mu - \delta\mathrm{i}_{\mathrm{GME}}^\mu, \mathrm{i}_\alpha^\mu + \delta\mathrm{i}_{\mathrm{GME}}^\mu]$ (i^0 is defined as the energy), and $\mathrm{I}_{\mathscr{H}_{\{\delta\mathbf{i}_{\mathrm{GME}}\}}}$ is the identity matrix in this subspace. Moreover, the GETH predicts that the mutual eigenstates with the similar values of the conserved quantities should be locally close to each other. As a result, the steady state of any superposition of these mutual eigenstates within the windows all gives the same result, which shows the "universality" of the generalized thermalization. Then, we will demonstrate that the SOC system satisfies the GETH and exhibits the generalized thermalization, i.e., the steady state can be predicted by the GME state.

For a mutual eigenstate with momentum k_0 and energy $E_0^{u(d)} = \pm|E(k_0)|$, its reduced density matrix reads

$$\mathrm{Tr}_{\mathcal{B}}[(|\mathbf{n}_{k_0}^{u(d)}\rangle \otimes |k_0\rangle)(\langle\mathbf{n}_{k_0}^{u(d)}| \otimes \langle k_0|)] = \frac{1}{2}[\mathrm{I} \pm \mathbf{n}_{\mathcal{H}}(k_0)], \tag{S20}$$

where "±" denotes that the mutual eigenstate lies in the upper (lower) band. On the other hand, the reduced state can also be obtained by the ensemble of the right-hand side in Eq. (S19) as

$$\begin{aligned}
\rho_{\mathrm{GME}} &= \mathrm{Tr}_{\mathcal{B}}\left[\frac{\mathrm{I}_{\mathscr{H}_{\{\delta\mathbf{i}_{\mathrm{GME}}\}}}}{\dim(\mathscr{H}_{\{\delta\mathbf{i}_{\mathrm{GME}}\}})}\right] \\
&= \frac{1}{N_{\delta k_{\mathrm{GME}}}}\mathrm{Tr}_{\mathcal{B}}[\sum_{|k-k_0|<\delta k_{\mathrm{GME}}} (|\mathbf{n}_k^{u(d)}\rangle \otimes |k\rangle)(\langle\mathbf{n}_k^{u(d)}| \otimes \langle k|)] \\
&= \frac{1}{2}[\mathrm{I} \pm \frac{1}{N_{\delta k_{\mathrm{GME}}}} \sum_{|k-k_0|<\delta k_{\mathrm{GME}}} \mathbf{n}_{\mathcal{H}}(k) \cdot \vec{\sigma}] \\
&\approx \frac{1}{2}[\mathrm{I} \pm \mathbf{n}_{\mathcal{H}}(k_0) \cdot \vec{\sigma} \pm \frac{1}{2N_{\delta k_{\mathrm{GME}}}} \ddot{\mathbf{n}}_{\mathcal{H}}(k)|_{k=k_0} \cdot \vec{\sigma} \sum_{\delta k_{\mathrm{GME}}} (k-k_0)^2] \\
&\xrightarrow{\delta k_{\mathrm{GME}}\ \text{is small}} \frac{1}{2}[\mathrm{I} \pm \mathbf{n}_{\mathcal{H}}(k_0) \cdot \vec{\sigma}],
\end{aligned} \tag{S21}$$

where δk_{GME} denots the half-width of the momentum window of the GME state (at the same time, the energy window is determined based on the function $E(k)$), which should

be macroscopically small but can cover enough mutual eigenstates, $\ddot{A}$ denotes the second derivative of A with respect to k, and $N_{\delta k_{\rm GME}}$ is the number of the mutual eigenstates whose momentum located in the momentum window. Since the GME state should be independent of the width of the chosen window [8], it satisfies the condition

$$\frac{\frac{1}{2}|\ddot{\mathbf{n}}_{\mathcal{H}}(k)|_{k=k_0}|\Delta^2_{k_{\rm GME}}}{|\mathbf{n}_{\mathcal{H}}(k_0)|} << 1, \tag{S22}$$

where $\Delta^2_{k_{\rm GME}} = \frac{1}{N_{\delta k_{\rm GME}}} \sum_{|k-k_0|<\delta k_{\rm GME}} (k-k_0)^2$, and $\Delta_{k_{\rm GME}}$ is the momentum standard deviation of the GME state. Obviously, the state in Eq. (S20) and Eq. (S21) are the same, indicating the validity of the GETH.

We further consider the superposition state of the mutual eigenstates with the similar conserved quantities k_0 and $E_0^{u(d)}$ (i.e., the momentum of these eigenstates lie in the window $[k_0-\delta k, k_0+\delta k]$, and their energies lie in the window $[E_0-\delta E_k, E_0+\delta E_k]$) (see Fig. S2) as

$$|\Psi(0)\rangle = \sum_{|k-k_0|<\delta k} e^{i\omega(k)}|\mathbf{n}_k^{u(d)}\rangle \otimes \sqrt{\mathcal{P}(k)}|k\rangle, \tag{S23}$$

where $\omega(k)$ denotes the phase.

According to Eq. (S4), the initial state finally relaxes to a steady state as

$$\begin{aligned}\rho_{\rm st} &= \sum_{|k-k_0|<\delta k} \frac{1}{2}[\mathrm{I} + \mathcal{P}(k)(\mathbf{n}_i(k)\cdot\mathbf{n}_{\mathcal{H}}(k))\mathbf{n}_{\mathcal{H}}(k)\cdot\vec{\sigma}] \\ &= \sum_{|k-k_0|<\delta k} \frac{1}{2}[\mathrm{I} \pm \mathcal{P}(k)\mathbf{n}_{\mathcal{H}}(k)\cdot\vec{\sigma}] \\ &\xrightarrow{\rm GETH} \frac{1}{2}[\mathrm{I} \pm \mathbf{n}_{\mathcal{H}}(k_0)\cdot\vec{\sigma}]. \end{aligned} \tag{S24}$$

Clearly, the steady state is only determined by the conserved quantities k_0 and $E(k_0)$ ($\mathcal{H}(k)$ is determined by k and $E(k)$). Moreover, the momentum standard deviation of the initial state Δ_k (for the Gaussian initial state adopted in the QW experiment, $\delta k = \sqrt{2\ln(2)}\Delta_k$) should satisfy Eq. (S22) to make sure the small conserved quantities fluctuation. Thus, the maximal value of can be estimated by the maximum value of $\Delta_{k_{\rm GME}}$ satisfying Eq. (S22). If the Δ_k (and the associated δk) of the initial state satisfies this condition, then its steady state is identical to the prediction of the GME.

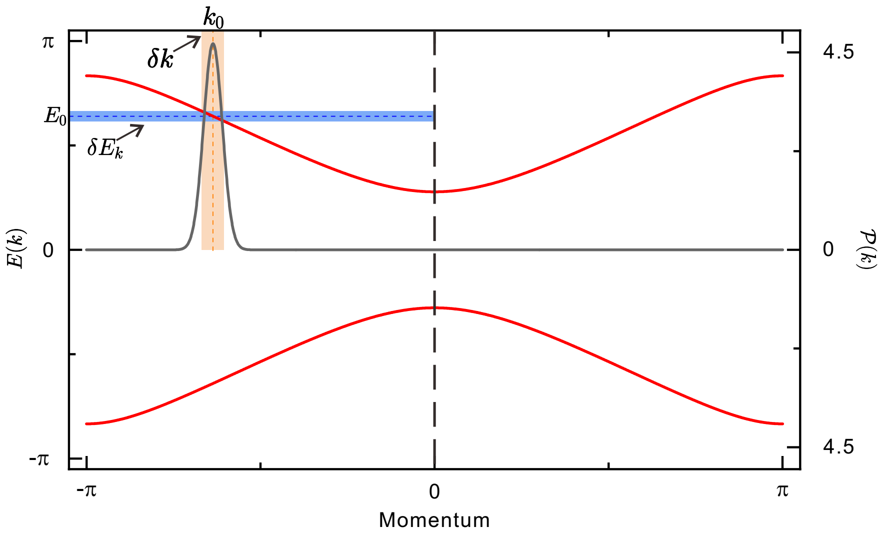

FIG. S2. Diagram of the initial state within a small energy-momentum window. The red line is the energy band of the SSQW effective Hamiltonian $\mathcal{H}_{\text{eff}}(\frac{4\pi}{9}, \frac{\pi}{9})$ [6], the orange region is the small momentum window $[k_0 - \delta k, k_0 + \delta k]$, and the blue region is its corresponding energy window $[E_0 - \delta E_k, E_0 + \delta E_k]$, where δE_k is determined by δk. We choose herein that the energy window is in upper energy band, so $E_0 > 0$. Moreover, there is only one part of the mutual eigenstates within this energy-momentum window with respect to the case in Fig. S1.

E. THE GENERALIZED THERMALIZATION IN EXTENDED SITUATIONS

As discussed in section C, due to the validity of the GETH, any superposition state of the mutual eigenstate within a small connected window exhibits the generalized thermalization. Further, we consider a superposition state of the mutual eigenstates within two separated windows and show the occurrence of the generalized thermalization in this extended situation can also be understood by the GETH.

Firstly, we consider the situation that both the two separated energy-momentum windows are within the same momentum window but different energy windows (see Fig. S3). The initial state then takes the form

$$|\Psi(0)\rangle = \sum\nolimits_{|k-k_0|<\delta k} (a_k|\mathbf{n}_k^u\rangle + b_k|\mathbf{n}_k^d\rangle) \otimes \sqrt{\mathcal{P}(k)}|k\rangle, \tag{S25}$$

where δk denotes the half-width of the small momentum window. The initial state's expected energy is $E_0 = \sum_{|k-k_0|<\delta k} E(k)\mathcal{P}(k)(|a_k|^2 - |b_k|^2)$ and expected momentum is

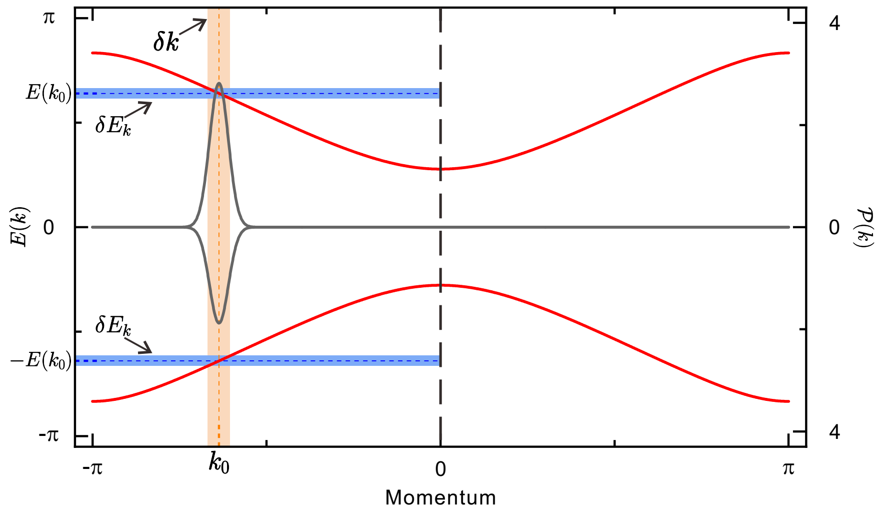

FIG. S3. Diagram of the initial state within two separated energy-momentum windows. The red line is the energy band of the SSQW effective Hamiltonian $\mathcal{H}_{\text{eff}}(\frac{4\pi}{9}, \frac{\pi}{9})$ [6], the orange region is the small momentum window $[k_0 - \delta k, k_0 + \delta k]$, and the two separated blue regions are the corresponding energy windows $[E(k_0) - \delta E_k, E(k_0) + \delta E_k]$ and $[-E(k_0) - \delta E_k, -E(k_0) + \delta E_k]$, where δE_k is determined by δk.

$k_0 = \sum_{|k-k_0|<\delta k} k\,\mathcal{P}(k)$. In addition, when $a_k \to 0$ or $b_k \to 0$, it reduces to the scenario discussed in the section C. According to Eq. (S4), we have

$$
\begin{aligned}
\rho_{\text{st}} &= \sum_{|k-k_0|<\delta k} \frac{1}{2}[\mathrm{I} + \mathcal{P}(k)(\mathbf{n}_i(k)\cdot\mathbf{n}_{\mathcal{H}}(k))\mathbf{n}_{\mathcal{H}}(k)\cdot\vec{\sigma}] \\
&= \frac{1}{2}[\mathrm{I} + \sum_{|k-k_0|<\delta k} \mathcal{P}(k)\,|a_k|^2 \mathbf{n}_{\mathcal{H}}(k)\cdot\vec{\sigma} - \sum_{|k-k_0|<\delta k} \mathcal{P}(k)\,|b_k|^2 \mathbf{n}_{\mathcal{H}}(k)\cdot\vec{\sigma}] \\
&\xrightarrow{\text{GETH}} \frac{1}{2}[\mathrm{I} + \tilde{\mathcal{P}}_{\text{up}}\mathbf{n}_{\mathcal{H}}(k_0)\cdot\vec{\sigma} - \tilde{\mathcal{P}}_{\text{down}}\mathbf{n}_{\mathcal{H}}(k_0)\cdot\vec{\sigma}] \\
&\approx \frac{1}{2}[\mathrm{I} + \frac{E_0}{E(k_0)}\mathbf{n}_{\mathcal{H}}(k_0)\cdot\vec{\sigma}],
\end{aligned} \tag{S26}
$$

where $\tilde{\mathcal{P}}_{\text{up}} \equiv \sum_{|k-k_0|<\delta k} \mathcal{P}(k)\,|a_k|^2$ and $\tilde{\mathcal{P}}_{\text{down}} \equiv \sum_{|k-k_0|<\delta k} \mathcal{P}(k)\,|b_k|^2$ are the upper and lower band occupancy of the initial state, respectively. The approximation of the last line can be gotten by applying $E_0 \approx E(k_0)\sum_{|k-k_0|<\delta k} \mathcal{P}(k)(|a_k|^2 - |b_k|^2)$. Moreover, the GME state in this extended situation is the weighted average of the GME states in the individual

connected window, and takes the form

$$\begin{aligned}\rho_{\mathrm{GME}} &= \tilde{\mathcal{P}}_{\mathrm{up}}\mathrm{Tr}_{\mathcal{B}}\left[\frac{\mathrm{I}_{\mathscr{H}_{\{\delta\mathbf{i}_{\mathrm{GME1}}\}}}}{\dim(\mathscr{H}_{\{\delta\mathbf{i}_{\mathrm{GME1}}\}})}\right] + \tilde{\mathcal{P}}_{\mathrm{down}}\mathrm{Tr}_{\mathcal{B}}\left[\frac{\mathrm{I}_{\mathscr{H}_{\{\delta\mathbf{i}_{\mathrm{GME2}}\}}}}{\dim(\mathscr{H}_{\{\delta\mathbf{i}_{\mathrm{GME2}}\}})}\right] \\ &\approx \frac{\tilde{\mathcal{P}}_{\mathrm{up}}}{2}[\mathrm{I} + \mathbf{n}_{\mathcal{H}}(k_0)\cdot\vec{\sigma}] + \frac{\tilde{\mathcal{P}}_{\mathrm{down}}}{2}[\mathrm{I} - \mathbf{n}_{\mathcal{H}}(k_0)\cdot\vec{\sigma}] \\ &\approx \frac{1}{2}[\mathrm{I} + \frac{E_0}{E(k_0)}\mathbf{n}_{\mathcal{H}}(k_0)\cdot\vec{\sigma}],\end{aligned} \tag{S27}$$

where $\mathscr{H}_{\{\delta\mathbf{i}_{\mathrm{GME1}}\}}$ ($\mathscr{H}_{\{\delta\mathbf{i}_{\mathrm{GME2}}\}}$) denotes the Hilbert subspace spanned by the eigenstates located in the upper (lower) energy window (see Fig. S3), and $\mathrm{I}_{\mathscr{H}_{\{\delta\mathbf{i}_{\mathrm{GME1}}\}}}(\mathrm{I}_{\mathscr{H}_{\{\delta\mathbf{i}_{\mathrm{GME2}}\}}})$ denotes the identity matrix in this subspace. The weights are the probabilities to be found in the upper connected window $\tilde{\mathcal{P}}_{\mathrm{up}}$ and the lower one $\tilde{\mathcal{P}}_{\mathrm{down}}$. Thus, based on the GETH, the steady state in this extended situation can also be characterized by the GME prediction and consequently only depends on E_0 and k_0, exhibiting the generalized thermalization.

Furthermore, we consider the situation that both the two separated energy-momentum windows are within the same energy window but different momentum windows (see Fig. S4), which has been previously used in section B to indicate the failure of the ETH (and thermalization) in the SSQW system. The initial state then reads

$$|\Psi(0)\rangle = \sum_{\substack{k>0;\\ |k-k_{E_0}|<\delta k_E}} [e^{i\omega_r(k)}|\mathbf{n}_k^{u(d)}\rangle \otimes \sqrt{\mathcal{P}(k)}|k\rangle + e^{i\omega_l(k)}|\mathbf{n}_{-k}^{u(d)}\rangle \otimes \sqrt{\mathcal{P}(-k)}|-k\rangle], \tag{S28}$$

where $\omega_{l(r)}(k)$ denotes the phase, and $|k - k_{E_0}| < \delta k_E$ ($k_{E_0} > 0$) is the positive momentum window, which is determined by the chosen energy window. The initial expected energy is $E_0 = \pm[\sum_{\substack{k>0;\\ |k-k_{E_0}|<\delta k_E}} (\mathcal{P}(k)E(k) + \mathcal{P}(-k)E(-k))]$, and expected momentum is $k_0 = \sum_{\substack{k>0;\\ |k-k_{E_0}|<\delta k_E}} [\mathcal{P}(k)k + \mathcal{P}(-k)(-k)]$. According to the Eq. (S4), we have

$$\begin{aligned}\rho_{\mathrm{st}} &= \sum\nolimits_k \frac{1}{2}[\mathrm{I} + \mathcal{P}(k)(\mathbf{n}_i(k)\cdot\mathbf{n}_{\mathcal{H}}(k))\mathbf{n}_{\mathcal{H}}(k)\cdot\vec{\sigma}] \\ &= \frac{1}{2}[\mathrm{I} \pm \sum_{\substack{k>0;\\ |k-k_{E_0}|<\delta k_E}} \mathcal{P}(k)\,\mathbf{n}_{\mathcal{H}}(k)\cdot\vec{\sigma} \pm \sum_{\substack{k>0;\\ |k-k_{E_0}|<\delta k_E}} \mathcal{P}(-k)\,\mathbf{n}_{\mathcal{H}}(-k)\cdot\vec{\sigma}] \\ &\xrightarrow{\mathrm{GETH}} \frac{1}{2}[\mathrm{I} \pm \tilde{\mathcal{P}}_{\mathrm{R}}\,\mathbf{n}_{\mathcal{H}}(k_{E_0})\cdot\vec{\sigma} \pm \tilde{\mathcal{P}}_{\mathrm{L}}\,\mathbf{n}_{\mathcal{H}}(-k_{E_0})\cdot\vec{\sigma}] \\ &\approx \frac{1}{2}[\mathrm{I} \pm \frac{k_{E_0}+k_0}{2k_{E_0}}\mathbf{n}_{\mathcal{H}}(k_{E_0})\cdot\vec{\sigma} \pm \frac{k_{E_0}-k_0}{2k_{E_0}}\mathbf{n}_{\mathcal{H}}(-k_{E_0})\cdot\vec{\sigma}],\end{aligned}$$

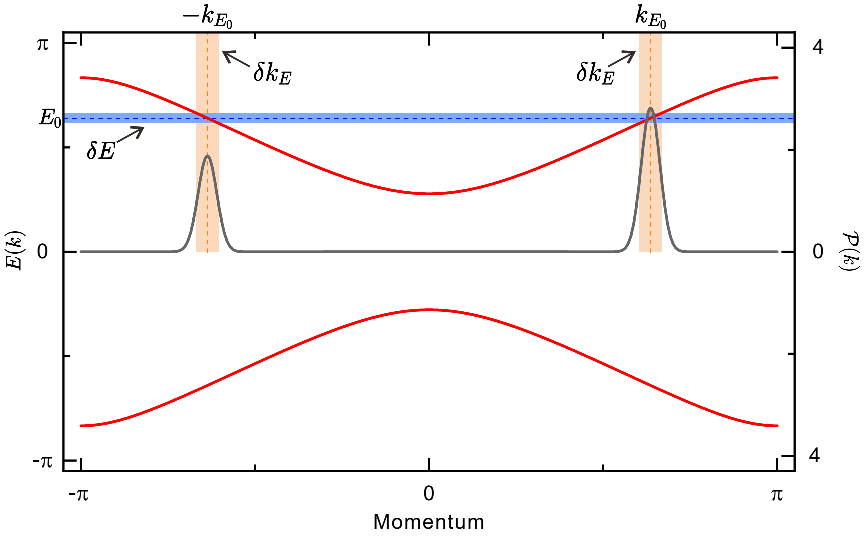

FIG. S4. Diagram of the initial state within two separated energy-momentum windows. The red line is the energy band of the SSQW effective Hamiltonian $\mathcal{H}_{\text{eff}}(\frac{4\pi}{9}, \frac{\pi}{9})$ [6], the blue region is the small energy window $[E_0 - \delta E, E_0 + \delta E]$, and the two separated orange regions are the corresponding momentum windows $[-k_{E_0} - \delta k_E, -k_{E_0} + \delta k_E]$ and $[k_{E_0} - \delta k_E, k_{E_0} + \delta k_E]$, where δk_E is determined by δE.

where $\tilde{\mathcal{P}}_{\rm R} = \sum_{\substack{k>0;\\ |k-k_{E_0}|<\delta k_E}} \mathcal{P}(k)$ and $\tilde{\mathcal{P}}_{\rm L} = \sum_{\substack{k>0;\\ |k-k_{E_0}|<\delta k_E}} \mathcal{P}(-k)$ $(k > 0)$ are the probabilities to be found in the right and left connected windows, respectively. The last approximation is obtained by applying $k_0 \approx k_{E_0} \sum_{\substack{k>0;\\ |k-k_{E_0}|<\delta k_E}} (\mathcal{P}(k) - \mathcal{P}(-k))$. Moreover, the GME state in this extended situation can also be the weighted average of the GME states in the right and left connected windows (the weights are $\tilde{\mathcal{P}}_{\rm R}$ and $\tilde{\mathcal{P}}_{\rm L}$), and takes the form

$$\begin{aligned} \rho_{\rm GME} &= \tilde{\mathcal{P}}_{\rm R} \mathrm{Tr}_{\mathcal{B}} \left[\frac{\mathrm{I}_{\mathscr{H}_{\{\delta \mathbf{i}'_{\rm GME1}\}}}}{\dim(\mathscr{H}_{\{\delta \mathbf{i}'_{\rm GME1}\}})} \right] + \tilde{\mathcal{P}}_{\rm L} \mathrm{Tr}_{\mathcal{B}} \left[\frac{\mathrm{I}_{\mathscr{H}_{\{\delta \mathbf{i}'_{\rm GME2}\}}}}{\dim(\mathscr{H}_{\{\delta \mathbf{i}'_{\rm GME2}\}})} \right] \\ &\approx \frac{\tilde{\mathcal{P}}_{\rm R}}{2} [\mathrm{I} \pm \mathbf{n}_{\mathcal{H}}(k_{E_0}) \cdot \vec{\sigma}] + \frac{\tilde{\mathcal{P}}_{\rm L}}{2} [\mathrm{I} \pm \mathbf{n}_{\mathcal{H}}(-k_{E_0}) \cdot \vec{\sigma}] \\ &\approx \frac{1}{2} [\mathrm{I} \pm \frac{k_{E_0} + k_0}{2k_{E_0}} \mathbf{n}_{\mathcal{H}}(k_{E_0}) \cdot \vec{\sigma} \pm \frac{k_{E_0} - k_0}{2k_{E_0}} \mathbf{n}_{\mathcal{H}}(-k_{E_0}) \cdot \vec{\sigma}], \end{aligned} \tag{S29}$$

where $\mathscr{H}_{\{\delta \mathbf{i}'_{\rm GME1}\}}$ ($\mathscr{H}_{\{\delta \mathbf{i}'_{\rm GME2}\}}$) is the Hilbert subspace spanned by the eigenstates located in the right (left) window (see Fig. S4), and $\mathrm{I}_{\mathscr{H}_{\{\delta \mathbf{i}'_{\rm GME1}\}}}$ ($\mathrm{I}_{\mathscr{H}_{\{\delta \mathbf{i}'_{\rm GME2}\}}}$) is the identity matrix in this subspace. As discussed in the section B, the SSQW system in this extended situation cannot be thermalized, and only with the energy E_0 is not enough to characterize its steady state; however, with another information of the conserved quantity–momentum k_0, the steady state can be predicated by the GME state in Eq. (S29), which is explicable by using the GETH.

F. RELATION WITH SU-SCHRIEFFER-HEEGER MODEL

In this part, we will show that the validity of GETH in SOC system will lead to a special case of the generalized thermaliztion in an equivalent many-body system like the Su-Schrieffer-Heeger (SSH) model[15]. For the SSH model, its Hamiltonian reads[16]

$$\mathcal{H}_{\mathrm{SSH}} = \sum_k \mathbf{c'}^{\dagger}_k \mathcal{H}_k \mathbf{c'}_k \tag{S30}$$

with $\mathbf{c'}_k = (c'_{s_1,k}, c'_{s_2,k})$. $c'_{i,k}$ $(i = s_1, s_2)$ are the annihilation operators in momentum space for two different sublattice in SSH model, and $\mathcal{H}_k = E(k)\mathbf{n}_{\mathcal{H}}(k) \cdot \vec{\sigma}$ where $E(k)$ and $\mathbf{n}_{\mathcal{H}}(k)$ is what we defined in the SOC system. By performing an unitary transformation for each individual $\mathcal{H}_k$,

$$U = \begin{pmatrix} \xi_{1,k} & \xi_{2,k} \\ -\xi^*_{2,k} & \xi^*_{1,k} \end{pmatrix} \tag{S31}$$

we can rewrite the Hamiltonian as

$$\mathcal{H}_{\mathrm{SSH}} = \sum_k (E(k)c^{\dagger}_{u,k}c_{u,k} - E(k)c^{\dagger}_{d,k}c_{d,k}) \tag{S32}$$

where $c_{u,k} = \xi^*_{1,k}c'_{s_1,k} - \xi_{2,k}c'_{s_2,k}$, and $c_{d,k} = \xi^*_{2,k}c'_{s_1,k} + \xi_{1,k}c'_{s_2,k}$. The energy eigenstate thus reads

$$|e_{\mathbf{M}}\rangle = \Pi^P_{k\in[-\pi,\pi]} M_k|0\rangle \tag{S33}$$

with $|0\rangle$ is vacuum state, which means there is no quasi-particle in the system. $\mathbf{M} \equiv \{M_k | M_k = c^{\dagger}_{u,k} \,\mathrm{or}\, c^{\dagger}_{d,k} \,\mathrm{or}\, I_k, k \in [-\pi,\pi]\}$ where I_k means that the eigenstate has no quasi-particle with momentum k, and P represents a permutation of M_k. As an example, considering a two quasi-particles state, where the momentum of the quasi-particles are k_1 and k_2, the permutation of M_k is two, the first one reads $|e_{\mathbf{M}_{1,2}}\rangle = c^{\dagger}_{u,k_1}c^{\dagger}_{u,k_2}|0\rangle = |1_{k_1}1_{k_2}\rangle = \frac{1}{\sqrt{2}}(|1_{k_1}\rangle|1_{k_2}\rangle - |1_{k_2}\rangle|1_{k_1}\rangle)$, and the second one reads $|e_{\mathbf{M}_{1,2}}\rangle = c^{\dagger}_{u,k_2}c^{\dagger}_{u,k_1}|0\rangle = |1_{k_2}1_{k_1}\rangle = \frac{1}{\sqrt{2}}(|1_{k_2}\rangle|1_{k_1}\rangle - |1_{k_1}\rangle|1_{k_2}\rangle)$. Obviously, these two states are not independent. Actually, it is

enough to only choose one permutation to generate a complete set of eigenbasis. In the following, for the energy eigenbasis we use, we choose the momentum sequential permutation. Namely, $e_{\mathbf{M}} = (M_{k_1} M_{k_2} ... M_{k_i} ...)|0\rangle$ where $k_1 < k_2 < ... < k_i < ....$

As we know, the Hermitian operator for a single spinor reads $o_0 \mathrm{I} + \vec{o} \cdot \vec{\sigma}$ where $\vec{o} = (o_1, o_2, o_3)$ is a vector. Now, considering the one-body observable with a specific momentum in this system, there are four kinds (In the following, we ignore the trivial parts of the observable, i.e. the terms proportional to the identity matrix). The first kind of the one-body observable reads $O_{1,k} = \mathbf{c}_{1,k}^{\dagger} (\vec{o}_{1,k} \cdot \vec{\sigma}_k) \mathbf{c}_{1,k}$ where $\mathbf{c}_{1,k} = (c_{u,k}, c_{d,k})$, and $\vec{\sigma}_k$ is the Pauli matrices in a new coordinate whose z-axis is parallel with the $\mathbf{n}_{\mathcal{H}}(k)$. In other word,

$$\begin{cases} \sigma_{x,k} = |\mathbf{n}_k^u\rangle\langle\mathbf{n}_k^d| + |\mathbf{n}_k^d\rangle\langle\mathbf{n}_k^u| \\ \sigma_{y,k} = -i|\mathbf{n}_k^u\rangle\langle\mathbf{n}_k^d| + i|\mathbf{n}_k^d\rangle\langle\mathbf{n}_k^u| \\ \sigma_{z,k} = |\mathbf{n}_k^u\rangle\langle\mathbf{n}_k^u| - |\mathbf{n}_k^d\rangle\langle\mathbf{n}_k^d| \end{cases} \tag{S34}$$

and $\vec{o}_{1,k} = (\vec{o}_{1,k} \cdot \vec{\sigma}_{x,k}, \vec{o}_{1,k} \cdot \vec{\sigma}_{y,k}, \vec{o}_{1,k} \cdot \vec{\sigma}_{z,k})$. Besides, the eigenstate expectation value of the first kind of one-body observable is

$$\langle e|O_{1,k_\alpha}|e\rangle = \begin{cases} \vec{o}_{1,k} \cdot \vec{\sigma}_{z,k} & M_{k_\alpha} = c_{u,k_\alpha} \\ -\vec{o}_{1,k} \cdot \vec{\sigma}_{z,k} & M_{k_\alpha} = c_{d,k_\alpha} \\ 0 & M_{k_\alpha} = I_{k_\alpha} \end{cases} \tag{S35}$$

The second kind of one-body observable reads $O_{2,k} = \mathbf{c}_{2,k}^{\dagger} (\vec{O}_{2,k} \cdot \vec{\sigma}_k) \mathbf{c}_{2,k}$ where $\mathbf{c}_{2,k} = (c_{u,k}^{\dagger}, c_{d,k})$, whose eigenstate expectation value is

$$\langle e|O_{2,k_\alpha}|e\rangle = \begin{cases} 0 & M_{k_\alpha} = c_{u,k_\alpha} \\ 0 & M_{k_\alpha} = c_{d,k_\alpha} \\ \vec{o}_{1,k} \cdot \vec{\sigma}_{z,k} & M_{k_\alpha} = I_{k_\alpha} \end{cases} \tag{S36}$$

The third kind of one-body observable reads $O_{3,k} = \mathbf{c}_{3,k}^{\dagger} (\vec{O}_{3,k} \cdot \vec{\sigma}_k) \mathbf{c}_{3,k}$ where $\mathbf{c}_{3,k} = (c_{u,k}, c_{d,k}^{\dagger})$, whose eigenstate expectation value is

$$\langle e|O_{3,k_\alpha}|e\rangle = \begin{cases} 0 & M_{k_\alpha} = c_{u,k_\alpha} \\ 0 & M_{k_\alpha} = c_{d,k_\alpha} \\ -\vec{o}_{1,k} \cdot \vec{\sigma}_{z,k} & M_{k_\alpha} = I_{k_\alpha} \end{cases} \tag{S37}$$

The forth kind of one-body observable reads $O_{4,k} = \mathbf{c}_{4,k}^{\dagger} (\vec{O}_{4,k} \cdot \vec{\sigma}_k) \mathbf{c}_{4,k}$ where $\mathbf{c}_{4,k} = (c_{u,k}^{\dagger}, c_{d,k}^{\dagger})$,

whose eigenstate expectation value is

$$\langle e|O_{4,k_\alpha}|e\rangle = \begin{cases} -\vec{o}_{1,k}\cdot\vec{\sigma}_{z,k} & M_{k_\alpha}=c_{u,k_\alpha} \\ \vec{o}_{1,k}\cdot\vec{\sigma}_{z,k} & M_{k_\alpha}=c_{d,k_\alpha} \\ 0 & M_{k_\alpha}=I_{k_\alpha} \end{cases} \tag{S38}$$

Furthermore, if the initial state $|\phi_0\rangle = \sum_j l_{\mathbf{M}_j}|e_{\mathbf{M}_j}\rangle$ with mean energy E_0 and mean momentum k_0 only occupies the energy eigenstates whose quasi-particle momentum is within a small momentum window $W_k = [\widetilde{k}_0 - \delta k, \widetilde{k}_0 + \delta k]$, which is similar with the case in the SOC system we have showed, and a one-body observable reads

$$\mathcal{O} = q_1 \sum_{k_\alpha\in W_k} O_{1,k_\alpha} + q_2 \sum_{k_\alpha\in W_k} O_{2,k_\alpha} + q_3 \sum_{k_\alpha\in W_k} O_{3,k_\alpha} + q_4 \sum_{k_\alpha\in W_k} O_{4,k_\alpha}. \tag{S39}$$

where $O_{1,k_\alpha}\cdot\mathbf{n}_{\mathcal{H}}(\widetilde{k}_0) = O_{1,\widetilde{k}_0}\cdot\mathbf{n}_{\mathcal{H}}(\widetilde{k}_0) = C_{k_0,1}$, $O_{2,k_\alpha}\cdot\mathbf{n}_{\mathcal{H}}(\widetilde{k}_0) = O_{2,\widetilde{k}_0}\cdot\mathbf{n}_{\mathcal{H}}(\widetilde{k}_0) = C_{k_0,2}$, $O_{3,k_\alpha}\cdot\mathbf{n}_{\mathcal{H}}(\widetilde{k}_0) = O_{3,\widetilde{k}_0}\cdot\mathbf{n}_{\mathcal{H}}(\widetilde{k}_0) = C_{k_0,3}$, $O_{4,k_\alpha}\cdot\mathbf{n}_{\mathcal{H}}(\widetilde{k}_0) = O_{4,\widetilde{k}_0}\cdot\mathbf{n}_{\mathcal{H}}(\widetilde{k}_0) = C_{k_0,4}$ for all k_α, then the expectation of $\mathcal{O}$ respecting to the diagonal ensemble of this initial state would be

$$\begin{aligned}
\mathrm{Tr}[\rho_{\mathrm{DE}}\mathcal{O}] =& q_1(\sum_{k_\alpha\in W_k}\sum_{\substack{j\\ M_{j,k_\alpha}=c_{u,k_\alpha}}} |l_{\mathbf{M}_j}|^2 O_{1,k_\alpha}\cdot\mathbf{n}_{\mathcal{H}}(k_\alpha) - \sum_{k_\alpha\in W_k}\sum_{\substack{j\\ M_{j,k_\alpha}=c_{d,k_\alpha}}} |l_{\mathbf{M}_j}|^2 O_{1,k_\alpha}\cdot\mathbf{n}_{\mathcal{H}}(k_\alpha)) \qquad \text{(S40)}\\
&+ q_2 \sum_{k_\alpha\in W_k}\sum_{\substack{j\\ M_{j,k_\alpha}=I_{k_\alpha}}} |l_{\mathbf{M}_j}|^2 O_{2,k_\alpha}\cdot\mathbf{n}_{\mathcal{H}}(k_\alpha) + q_3(-\sum_{k_\alpha\in W_k}\sum_{\substack{j\\ M_{j,k_\alpha}=I_{k_\alpha}}} |l_{\mathbf{M}_j}|^2 O_{3,k_\alpha}\cdot\mathbf{n}_{\mathcal{H}}(k_\alpha))\\
&+ q_4(-\sum_{k_\alpha\in W_k}\sum_{\substack{j\\ M_{j,k_\alpha}=c_{u,k_\alpha}}} |l_{\mathbf{M}_j}|^2 O_{4,k_\alpha}\cdot\mathbf{n}_{\mathcal{H}}(k_\alpha) + \sum_{k_\alpha\in W_k}\sum_{\substack{j\\ M_{j,k_\alpha}=c_{d,k_\alpha}}} |l_{\mathbf{M}_j}|^2 O_{4,k_\alpha}\cdot\mathbf{n}_{\mathcal{H}}(k_\alpha))\\
\approx& q_1(\sum_{k_\alpha\in W_k}\sum_{\substack{j\\ M_{j,k_\alpha}=c_{u,k_\alpha}}} |l_{\mathbf{M}_j}|^2 O_{1,k_\alpha}\cdot\mathbf{n}_{\mathcal{H}}(\widetilde{k}_0) - \sum_{k_\alpha\in W_k}\sum_{\substack{j\\ M_{j,k_\alpha}=c_{d,k_\alpha}}} |l_{\mathbf{M}_j}|^2 O_{1,k_\alpha}\cdot\mathbf{n}_{\mathcal{H}}(\widetilde{k}_0))\\
&+ q_2 \sum_{k_\alpha\in W_k}\sum_{\substack{j\\ M_{j,k_\alpha}=I_{k_\alpha}}} |l_{\mathbf{M}_j}|^2 O_{2,k_\alpha}\cdot\mathbf{n}_{\mathcal{H}}(\widetilde{k}_0) + q_3(-\sum_{k_\alpha\in W_k}\sum_{\substack{j\\ M_{j,k_\alpha}=I_{k_\alpha}}} |l_{\mathbf{M}_j}|^2 O_{3,k_\alpha}\cdot\mathbf{n}_{\mathcal{H}}(\widetilde{k}_0))\\
&+ q_4(-\sum_{k_\alpha\in W_k}\sum_{\substack{j\\ M_{j,k_\alpha}=c_{u,k_\alpha}}} |l_{\mathbf{M}_j}|^2 O_{4,k_\alpha}\cdot\mathbf{n}_{\mathcal{H}}(\widetilde{k}_0) + \sum_{k_\alpha\in W_k}\sum_{\substack{j\\ M_{j,k_\alpha}=c_{d,k_\alpha}}} |l_{\mathbf{M}_j}|^2 O_{4,k_\alpha}\cdot\mathbf{n}_{\mathcal{H}}(\widetilde{k}_0))\\
=& (q_1 C_{\widetilde{k}_0,1} - q_4 C_{\widetilde{k}_0,4})L_u + (q_4 C_{\widetilde{k}_0,4} - q_1 C_{\widetilde{k}_0,1})L_d + (q_2 C_{\widetilde{k}_0,1} - q_3 C_{\widetilde{k}_0,4})L_0
\end{aligned}$$

where $L_u \equiv \sum_{k_\alpha\in W_k}\sum_{\substack{j\\ M_{j,k_\alpha}=c_{u,k_\alpha}}} |l_{\mathbf{M}_j}|^2$, $L_d \equiv \sum_{k_\alpha\in W_k}\sum_{\substack{j\\ M_{j,k_\alpha}=c_{d,k_\alpha}}} |l_{\mathbf{M}_j}|^2$, and $L_0 \equiv \sum_{k_\alpha\in W_k}\sum_{\substack{j\\ M_{j,k_\alpha}=I_{k_\alpha}}} |l_{\mathbf{M}_j}|^2 = N_{W_k} - L_u - L_d$ with $N_{W_k} = \sum_{k_\alpha\in W_k} 1$. We can see the key part of the derivation above is

the approximation. Namely, we can replace all $\mathbf{n}_{\mathcal{H}}(k_\alpha)$, where k_α is within the momentum window, by $\mathbf{n}_{\mathcal{H}}(\widetilde{k}_0)$. This is due to the validity of the GETH in the SOC system.

Moreover, considering the conserved charges of the initial state, i.e. the energy and the momentum, we have

$$\begin{aligned} E_0 &= \sum_{k_\alpha \in W_k} \sum_{\substack{j \\ M_{j,k_\alpha}=c_{u,k_\alpha}}} E(k)|l_{\mathbf{M}_j}|^2 - \sum_{k_\alpha \in W_k} \sum_{\substack{j \\ M_{j,k_\alpha}=c_{d,k_\alpha}}} E(k)|l_{\mathbf{M}_j}|^2 \qquad \text{(S41)} \\ &\approx E(\widetilde{k}_0)(L_u - L_d), \\ k_0 &= \sum_{k_\alpha \in W_k} \sum_{\substack{j \\ M_{j,k_\alpha}=c_{u,k_\alpha}}} k|l_{\mathbf{M}_j}|^2 - \sum_{k_\alpha \in W_k} \sum_{\substack{j \\ M_{j,k_\alpha}=c_{d,k_\alpha}}} k|l_{\mathbf{M}_j}|^2 \\ &\approx \widetilde{k}_0(L_u + L_d) \qquad \text{(S42)} \end{aligned}$$

where both two approximation is valid when W_k is small. We thus can get

$$L_u = \frac{1}{2}(\frac{k_0}{\widetilde{k}_0} + \frac{E_0}{E(\widetilde{k}_0)}), \qquad \text{(S43)}$$

$$L_d = \frac{1}{2}(\frac{k_0}{\widetilde{k}_0} - \frac{E_0}{E(\widetilde{k}_0)}). \qquad \text{(S44)}$$

Then, the expectation of the one-body operator we defined above is only dependent on three initial factors, i.e., the initial energy E_0, the initial momentum k_0, and the mean momentum of the momentum window $\widetilde{k}_0$. Thus, the generalized thermalization can occur in this special situation.

All above, it is the validity of GETH in SOC system that causes the satisfaction of the generalized thermalization in SSH model.

---

[1] P. Reimann, *Foundation of statistical mechanics under experimentally realistic conditions,* Phys. Rev. Lett. **101**, 190403 (2008).

[2] N. Linden, S. Popescu, A. J. Short, and A. Winter, *Quantum mechanical evolution towards thermal equilibrium,* Phys. Rev. E **79**, 061103 (2009).

[3] S. Trotzky, Y. A. Chen, A. Flesch, I. P. McCulloch, U. Schollwöck, J. Eisert, and I. Bloch, *Probing the relaxation towards equilibrium in an isolated strongly correlated one-dimensional bose gas,* Nat. Phys. **8**, 325 (2012).

[4] T. Langen, R. Geiger, M. Kuhnert, B. Rauer, and J. Schmiedmayer, *Local emergence of thermal correlations in an isolated quantum many-body system,* Nat. Phys. **9**, 640 (2013).

[5] C. Gogolin and J. Eisert, *Equilibration, thermalisation, and the emergence of statistical mechanics in closed quantum systems,* Rep. Prog. Phys. **79**, 056001 (2016).

[6] T. Kitagawa, M. S. Rudner, E. Berg, and E. Demler, *Exploring topological phases with quantum walks,* Phys. Rev. A **82**, 033429 (2010).

[7] Q.-Q. Wang, X.-Y. Xu, W.-W. Pan, S.-J. Tao, Z. Chen, Y.-T. Zhan, K. Sun, J.-S. Xu, G. Chen, Y.-J. Han, C.-F. Li, and G.-C. Guo, *Robustness of entanglement as an indicator of topological phases in quantum walks,* Optica **7**, 53–58 (2020).

[8] M. Rigol, V. Dunjko, and M. Olshanii, *Thermalization and its mechanism for generic isolated quantum systems,* Nature **452**, 854 (2008).

[9] M. Rigol, *Fundamental asymmetry in quenches between integrable and nonintegrable systems,* Phys. Rev. Lett. **116** (2016).

[10] L. P. García-Pintos, N. Linden, A. S. L. Malabarba, A. J. Short, and A. Winter, *Equilibration time scales of physically relevant observables,* Phys. Rev. X **7**, 031027 (2017).

[11] S. Goldstein, J. L. Lebowitz, R. Tumulka, and N. Zanghì, *Canonical typicality,* Phys. Rev. Lett. **96**, 050403 (2006).

[12] J. M. Deutsch, *Eigenstate thermalization hypothesis,* Rep. Prog. Phys. **81** (2018).

[13] A. C. Cassidy, C. W. Clark, and M. Rigol, *Generalized thermalization in an integrable lattice system,* Phys. Rev. Lett. **106**, 140405 (2011).

[14] T. Mori, T. N. Ikeda, E. Kaminishi, and M. Ueda, *Thermalization and prethermalization in isolated quantum systems: a theoretical overview,* J. Phys. B: At., Mol. Opt. Phys. **51**, 112001 (2018).

[15] W. P. Su, J. R. Schrieffer, and A. J. Heeger, *Solitons in polyacetylene,* Phys. Rev. Lett. **42**, 1698 (1979).

[16] J. K. Asbóth, L. Oroszlány, and A. Pályi, The su-schrieffer-heeger (ssh) model, in *A Short Course on Topological Insulators: Band Structure and Edge States in One and Two Dimensions* (Springer International Publishing, Cham, 2016) pp. 1–22.
